# Computational Insights into the Interaction of the Conserved Cysteine-Noose Domain of the Human Respiratory Syncytial Virus G Protein with the Canonical Fractalkine Binding site of Transmembrane Receptor CX3CR1 Isoforms

**DOI:** 10.3390/membranes14040084

**Published:** 2024-04-04

**Authors:** João Victor Piloto, Raphael Vinicius Rodrigues Dias, Wan Suk Augusto Mazucato, Marcelo Andres Fossey, Fernando Alves de Melo, Fabio Ceneviva Lacerda Almeida, Fatima Pereira de Souza, Icaro Putinhon Caruso

**Affiliations:** 1 Multiuser Center for Biomolecular Innovation (CMIB), Department of Physics, São Paulo State University (UNESP), São Jose do Rio Preto 15054-000, Brazil; joao.piloto@unesp.br (J.V.P.); rvr.dias@unesp.br (R.V.R.D.); wan.mazucato@unesp.br (W.S.A.M.); marcelo.fossey@unesp.br (M.A.F.); fernando.melo@unesp.br (F.A.d.M.); fatima.p.souza@unesp.br (F.P.d.S.); 2 Institute of Medical Biochemistry (IBqM), National Center of Nuclear Magnetic Resonance Jiri Jonas, Federal University of Rio de Janeiro, Rio de Janeiro 21941-902, Brazil; falmeida@bioqmed.ufrj.br

**Keywords:** CX3CR1, hRSV G protein, fractalkine binding site, computational tools

## Abstract

The human Respiratory Syncytial Virus (hRSV) stands as one of the most common causes of acute respiratory diseases. The infectivity of this virus is intricately linked to its membrane proteins, notably the attachment glycoprotein (G protein). The latter plays a key role in facilitating the attachment of hRSV to respiratory tract epithelial cells, thereby initiating the infection process. The present study aimed to characterize the interaction of the conserved cysteine-noose domain of hRSV G protein (cndG) with the transmembrane CX3C motif chemokine receptor 1 (CX3CR1) isoforms using computational tools of molecular modeling, docking, molecular dynamics simulations, and binding free energy calculations. From MD simulations of the molecular system embedded in the POPC lipid bilayer, we showed a stable interaction of cndG with the canonical fractalkine binding site in the N-terminal cavity of the CX3CR1 isoforms and identified that residues in the extracellular loop 2 (ECL2) region and Glu279 of this receptor are pivotal for the stabilization of CX3CR1/cndG binding, corroborating what was reported for the interaction of the chemokine fractalkine with CX3CR1 and its structure homolog US28. Therefore, the results presented here contribute by revealing key structural points for the CX3CR1/G interaction, allowing us to better understand the biology of hRSV from its attachment process and to develop new strategies to combat it.

## 1. Introduction

The human Respiratory Syncytial Virus (hRSV) is one of the major causes of acute respiratory diseases, primarily affecting children up to the age of five. This virus infects almost all children at least once by the age of two, with half of them experiencing at least two infections during this period [1,2]. Worldwide, approximately 33 million cases of illness occur in children annually, leading to around 3.2 million hospitalizations and nearly 200,000 deaths [3]. While the primary at-risk group comprises premature infants, immunodeficient individuals, and those with cardiopulmonary diseases, elderly and immunocompromised individuals are also part of the at-risk population [4,5]. This leads to a substantial impact, frequently reaching levels similar to those of influenza virus infections [6].

The symptoms of hRSV infection usually mimic a mild cold, including sneezing, nasal congestion, a runny nose, a sore throat, and fever. Nevertheless, in high-risk individuals, hRSV infection can rapidly advance to the lower respiratory tract, potentially resulting in the development of severe and potentially fatal conditions like pneumonia or severe pulmonary diseases. Despite recent progress with RSV vaccines for the elderly and pregnant women [7,8,9,10], there is still no effective treatment for severe hRSV cases [11,12]. In more severe cases involving compromised children, the recommended preventive treatment is the humanized monoclonal antibody Palivizumab targeted to the viral fusion protein [12]. However, access to this medication is significantly restricted due to its high cost. The only approved antiviral drug for hRSV treatment is aerosolized Ribavirin (used for treating other respiratory diseases), which is a guanine nucleotide analog targeting the viral polymerase. Ribavirin plays a significant role in the treatment of immunocompromised adults but is associated with some side effects [13].

In the hRSV viral envelope, there are attachment (G) and fusion (F) glycoproteins that project from the lipid bilayer on the surface of the virus [1]. Nowadays, current vaccines and most candidates for vaccines in preclinical and clinical development are primarily focused on triggering immune responses to the RSV F protein. This choice is driven by the characteristic of the F protein of being the most conserved across various strains/serotypes of the virus and its potential to induce a broader spectrum of protective immunity [14,15,16]. Nevertheless, the primary host/virus interaction originates from the binding of hRSV G protein with the transmembrane CX3C motif chemokine receptor 1 (CX3CR1) on the cellular surface of human lung cells [17,18,19], influencing the host’s immune response and aiding infection [20,21]. Unlike the broad set of structural data available for the hRSV F protein [22,23,24,25,26,27], the literature does not present significant structural information on the G protein or its binding to the transmembrane CX3C motif chemokine receptor 1 (CX3CR1), the primary contact in the host/virus interaction that stands out as a key point for understanding the biology of hRSV and for developing strategies to combat it.

There are still no structural data available on the interaction of the hRSV G protein with the transmembrane receptor CX3CR1. Structural data available in the literature report that the hRSV G protein is a type II membrane protein glycosylated with O- and N-linked glycans, which collectively contribute to approximately 60% of its molecular weight [26]. Its ectodomain comprises a central, conserved, non-glycosylated region flanked by two domains that lack a defined structure [26,27,28]. The central region contains 13 amino acid residues that display a high degree of conservation across all RSV strains, forming a cysteine noose with two disulfide bridges Cys173–Cys186 and Cys176–Cys182, thus establishing a motif for CX3C interaction similar to fractalkine, a CX3C motif chemokine ligand 1 (CX3CL1) [29].

The human transmembrane receptor CX3CR1 of the G protein-coupled receptor 1 family and the only known member of the CX3C chemokine receptor subfamily is found in four isoforms, differing in the number of N-terminal residues for which isoforms 2 and 4 show an additional 32 compared to isoform 1, the reference with 355 amino acid residues, while isoform 3 contains 7. It is worth noting that the main difference between isoforms 2 and 4 lies in the substitution of a lysine residue with phenylalanine in isoform 4 (Figure S1). Garin and collaborators reported that the CX3CR1 isoforms bound CX3CL1 with similar affinity but with on- and off-kinetic rate constants being significantly higher for the extended CX3CR1 isoforms, suggesting that the N-terminal extensions may alter the functions induced by CX3CL1. They also showed that the CX3CR1 isoforms with N-terminal extensions were slightly more sensitive than isoform 1 in functional assays such as calcium response and chemotaxis [30]. Recently, cryo-EM structures of human CX3CR1/heterotrimeric endogenous G-protein (Gi1) complexes in the ligand-free and CX3CL1-bound states were solved for transmembrane receptor isoform 1. Note the difference in intracellular G protein coupled to the CX3CR1 in the cryo-EM structure from the exogenous hRSV G protein, which is the focus of this study. The receptor CX3CR1 exhibits a barrel-like structure composed of a helical bundle with seven α-helices [31]. In the CX3CL1-bound state, the fractalkine is bound to the binding cavity in the N-terminal portion of the receptor CX3CR1, as well as for its structural homolog US28, a viral G protein-coupled receptor [31,32]. It is worth mentioning that the CX3CR1/CX3CL1 signaling axis also presents a key role in multiple neurodegenerative and inflammatory disorders as well as in tumorigenesis [33,34,35].

Here, we report structural insights into the interaction of the conserved cysteine-noose domain of hRSV G protein (cndG) with the transmembrane receptor CX3CR1 isoforms embedded in the POPC lipid bilayer, using computational tools such as molecular modeling, docking, molecular dynamics simulations, and binding free energy calculations. We modeled the four CX3CR1 isoforms in their free state and proposed the structural models of the complexes of these isoforms with the conserved cysteine-noose domain of the hRSV G protein, considering the fractalkine binding cavity in the N-terminal portion of the transmembrane receptor as the putative binding site for cndG. After a total of 2.4 μs of molecular dynamics (MD) simulations to evaluate the structural stability of the models of the CX3CR1/cndG complexes, the key amino acid residues for the interaction of CX3CR1 and cndG were presented and analyzed based on the proposed structural models.

## 2. Materials and Methods

### 2.1. Molecular Modeling of the Transmembrane Receptor CX3CR1 Isoforms

The molecular modeling of isoform 1 (UniProt ID P49238) of the cellular receptor CX3CR1 was performed using the standard protocol of the servers RoseTTAFold [36], trRosetta [37], AlphaFold [38], I-TASSER [39], and Phyre2 [40]. The structural evaluation of the CX3CR1 models was carried out from the Ramachandran plot using the program PROCHECK [41]. The structural models with more than 90% of the residues in the most favored regions of the Ramachandran plot were constructed using molecular dynamics simulations in a POPC lipid bilayer for the next evaluation step. After MD simulations, the protein structure prediction server of the best-evaluated model (from RoseTTAFold) was selected to construct the structure models of CX3CR1 isoforms 2, 3, and 4 (UniProt ID P49238-2, P49238-3, and P49238-4, respectively).

### 2.2. Molecular Docking of the cndG with CX3CR1 Isoforms

For molecular docking calculations, we used the servers ClusPro [42], HADDOCK [43], pyDockWEB [44], ZDOCK [45], HDOCK [46], HPEPDOCK [47], and MDockPeP [48], applying the standard protocol for each one. The structure of the conserved cysteine-noose domain of the hRSV G protein (cndG) was acquired from the PDB database under access code 5WN9 [49]. The representative structures of the CX3CR1 applied in the docking calculations were obtained from the clustering analysis performed for the 300 ns MD trajectory of the isoforms embedded in the POPC lipid bilayer. Using isoform 1 sequence numbering as a reference, the residues Glu174–Gly177 of the β-strand in the extracellular loop 2 (ECL2) at the entrance to the binding cavity in the N-terminal portion of the CX3CR1 were employed to drive the docking calculations. Note that isoforms 2 and 4 have 32 more residues in the N-terminal and isoform 3 only seven, changing the numbering of the residues in this β-strand. These residues of the ECL2 region were chosen based on their interactions established with fractalkine in the cryo-EM structure of a human CX3CR1/Gi1 complex in the CX3CL1-bound states (PDB id 7XBX) [31] and crystal structure of the CX3CL1 binding to the receptor US28 (PDB id 4XT1), a structural homolog of CX3CR1 with 38% sequence identity [32]. The ClusPro and HADDOCK servers were the top-ranked ones, and their resulting structural docking models were employed for the stages of production and evaluation of molecular dynamics simulations.

### 2.3. Molecular Dynamics Simulations of the CX3CR1/cndG Complexes

For the molecular dynamics (MD) simulation of the free and cndG-bound CX3CR1 isoforms in a lipid bilayer, the GROMOS 54A7 force field [50] was used with modifications to include topology parameters for lipids [51] and the TIP3P water model [52]. The CHARMM-GUI server [53] was applied to construct the lipid bilayer [54] using the phospholipid POPC (1-palmitoyl-2-oleoyl-sn-glycero-3-phosphocholine) in a rectangular box with the CX3CR1 aligned along the *Z*-axis of the bilayer. A water layer of 50 molecules per lipid with 22.5 Å thickness was added on both sides of the POPC bilayer. The lipid bilayer consisted of an average of 200 lipids in the upper layer and 200 lipids in the lower layer, totaling 400 lipids. The protonation state of ionizable CX3CR1 residues was determined using the PROPKA program [55], assuming a pH of 7.0. Sodium ions Na+ were added to neutralize the system within the rectangular box. Periodic boundary conditions were applied for the NPT ensemble, maintaining a constant temperature of 298 K and a pressure of 1.0 bar, with a Nose–Hoover thermostat (τ_T_ = 2 ps) and Parrinello–Rahman barostat (τ_P_ = 2 ps and compressibility = 4.5 × 10^–5^⋅bar^–1^). A cutoff of 12 Å was employed for the Lennard-Jones potential, and the Particle Mesh Ewald (PME) algorithm was used for the electrostatic potential calculations. Simulations were carried out using a time step of 2.0 fs, saving the trajectory every 20 ps, and all covalent bonds were constrained at their equilibrium distance. A total of 25 million steps (50 ns) were performed for each NPT (isothermal-isobaric) equilibration process of the system, applying a constant force of 1000 kJ·mol^–1^·nm^–2^ to all heavy atoms. Following the preparation steps, 300 ns MD simulations were conducted for the structural models of free and cndG-bound CX3CR1 isoforms embedded in the POPC lipid bilayer.

After MD simulations, the trajectories of the free and cndG-bound CX3CR1 isoforms were aligned and analyzed. For free isoforms in the POPC lipid bilayer, the following analysis was performed: root mean square deviation (RMSD) of the backbone atoms of CX3CR1; number of contacts <0.6 nm between the atoms of the residues Glu174–Gly177 of the ECL2 β-strand (numbering referring to isoform 1) in the canonical fractalkine binding site of the cavity and the N-terminal residues of CX3CR1 (isoform 1: residues 1–22; isoform 3: residues 1–31; and isoform 2 and 4: residues 1–51); and cluster analysis from the tool *g_cluster* [56] of the GROMACS package version 5.0.7 applying the *gromos* algorithm [57] with a cutoff of 2 Å for the determination of the representative structure of the models (employed in docking calculations) in the last 250 ns of the trajectories. The simulations of the structural models of the CX3CR1/cndG complexes were carried out in an analysis of the trajectories based on the RMSD of the backbone atoms of the isoforms; RMSD of the cndG relative to the CX3CR1 structure alignment; RMSD of the cndG relative to its own structure alignment; number of contacts < 0.6 nm between the atoms of the isoforms and of the cndG; distance calculations between the center of mass of the cndG and of the isoforms; cluster analysis from the tool *g_cluster* applying the *gromos* algorithm with a cutoff of 2 Å for the determination of the representative structures of the isoforms/cndG complexes; number of CX3CR1/cndG hydrogen bonds with a cutoff distance (heavy atoms) of 3.5 Å and maximum angle of 30°; percentages of persistence of the hydrogen bonds calculated from plot_hbmap_generic.pl script [58] and counted concerning residues of the isoforms and cndG for values greater than 10%; and the contributions of the residues of CX3CR1 and cndG to the theoretical Gibbs free energy of binding ($∆G_{b}$) calculated from the Molecular Mechanics Generalized Born Surface Area (MM-GBSA) method implemented in the *gmx_mmpbsa* program [59] for the GROMACS package, using 100 frames for the last 280 ns of simulation and applying a solute dielectric constant of 2, solvent dielectric constant of 80, NaCl concentration of 150 mM, and temperature of 298 K.

## 3. Results and Discussion

### 3.1. Molecular Modeling and MD Simulations of Structural Models of the CX3CR1 Isoforms Embedded in POPC Lipid Bilayer

**Figure 1:**
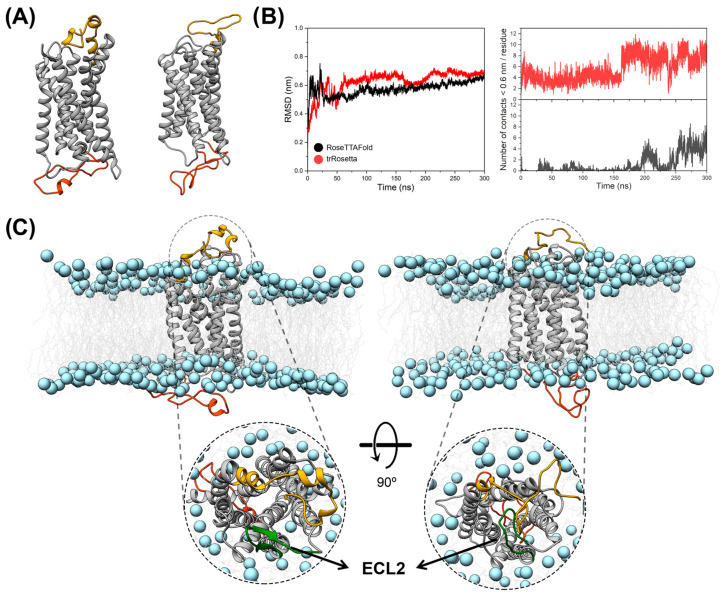
Tructural modeling of the transmembrane receptor CX3CR1 isoform 1. (**A**) On the left is the structure calculated by the RoseTTAFold server, and on the right by trRosetta. Both three-dimensional structures are represented as cartoons with the helical bundle denoted in grey, while the N-terminal and C-terminal regions are indicated in gold and orange, respectively. (**B**) Values of RMSD (**left**) and number of contacts <0.6 nm (**right**) obtained from the MD trajectories conducted with the structural models in A. Black color denotes the results for the RoseTTAFold model and red for the trRosetta model. (**C**) The most representative structure from clustering analysis for each MD trajectory of the structural model of the CX3CR1 isoform 1 embedded in the POPC lipid bilayer. The RoseTTAFold model is denoted on the left and the trRosetta model is on the right. The ECL2 region is denoted in green and indicated by the arrow. The proteins are represented as cartoons and the POPC lipids are indicated as light gray lines for their hydrophobic tails and as blue spheres for their phosphorus atoms.

Although the cryo-EM structure of CX3CR1 isoform 1 was recently published with resolutions of 2.8 and 3.4 Å [31], we employed molecular modeling tools to construct the structural models of the four isoforms in the free state, since the experimentally solved structure had 40 C-terminal residues truncated. It also involved the introduction of three mutations in the wild-type sequence to increase the efficiency of recombinant protein production. In addition, it is worth mentioning that the N-terminal residues did not have their structure solved experimentally, probably due to the inherent conformational flexibility of this region. Therefore, using the primary sequence of the cellular receptor CX3CR1 isoform 1, we explored all programs described in Section 2.1 for the evaluation of structural models. Initially, the first stage of assessing the ψ and ϕ angles was conducted using Ramachandran plots, where there were initial indications that the Rosetta tools were the most suitable for constructing molecular models, presenting more than 90% of residues in the most favored regions of this plot (Table S1). Figure 1A shows the structural models of CX3CR1 isoform 1, determined by RoseTTAFold and trRosetta, which present a barrel-like structure composed of a helical bundle with seven α-helices as seen for the cryo-EM structure solved recently [31]. The structural alignment for the residues of the helical bundle of these models with the cryo-EM structure revealed a significant similarity in their three-dimensional conformations, with RMSD < 1.6 Å (Figure S2). Subsequently, molecular dynamics (MD) simulations of 300 ns in the POPC lipid bilayer were performed for the three-dimensional structural models of the cellular receptor CX3CR1 isoform 1 to evaluate the structural stability of the models calculated by RoseTTAFold and trRosetta. Figure 1B shows the RMSD values calculated for the backbone atoms of CX3CR1 isoform 1 over 300 ns of simulation, employing the constructed model as the reference structure for the alignment. After 50 ns of simulation, it is possible to observe that the RMSD values reach stability levels for both structural models. It is noteworthy that for the model determined by RoseTTAFold, lower RMSD values were observed at the stability level than for the model calculated by trRosetta.

To verify the exposure of the binding cavity entrance of the cellular receptor CX3CR1 isoform 1 concerning the N-terminal region of the protein, the number of contacts < 0.6 nm was calculated between the atoms of the N-terminal residues Met1–Ile23 and those of Glu174–Gly177 of the ECL2 β-strand (numbers referring to isoform 1) at the entrance to the binding cavity throughout the 300 ns of simulation. As seen in Figure 1B, a greater number of contacts are observed for the structural model determined by trRosetta, which means that the binding pocket in the protein is occluded by the N-terminal residues for most of the simulation time. On the other hand, for the RoseTTAFold model, it is possible to observe reduced values for the number of contacts and a lower frequency of occurrence when compared to the trRosetta, indicating that the binding cavity is exposed for most of the simulation time. A clustering analysis of the trajectories of the 300 ns simulation of the structural models of CX3CR1 isoform 1 corroborates the analysis of the number of contacts, revealing that the binding cavity in the N-terminal portion (and notably ECL2) of the protein is exposed in the representative structure for the RoseTTAFold structural model (Figure 1C, left), whereas it is occluded for the trRosetta model (Figure 1C, right).

Based on the results of the Ramachandran plots and MD trajectory analyses for the structural models of CX3CR1 isoform 1, we proceeded with molecular modeling calculations for isoforms 2, 3, and 4 using the RoseTTAFold approach followed by MD simulations of 300 ns. As for isoform 1, the RMSD values for CX3CR1 isoforms 2, 3, and 4 reached stability levels after the 50 ns MD simulations (Figure S3). The number of contacts <0.6 nm between the atoms of the N-terminal residues and the β-strand of the ECL2 region (Glu174–Gly177, numbering referring to isoform 1) at the entrance to the binding cavity presents a sparse and reduced count like that observed for isoform 1, indicating that the binding cavity in the N-terminal portion of the protein is exposed, which corroborates the representative structures for CX3CR1 isoforms 2, 3, and 4 in the POPC lipid bilayer obtained by a clustering analysis of MD trajectories (Figure S4).

We also assessed the structural stability of the lipid bilayer by analyzing the mass density profiles for its different components. From the analysis of these profiles, it is possible to observe that the POPC lipid bilayer is stable throughout the 300 ns of simulation (Figure S5) since the nonpolar lipid chain occupies the central region of the Z coordinate axis (perpendicular to the lipid bilayer). Water molecules are found at the ends of the Z coordinate axis. The protein is internalized in the lipid bilayer, and phosphorus atoms occupy the threshold region between water molecules and nonpolar lipid atoms.

### 3.2. Docking and MD Simulations of the Interaction of cndG with the Canonical Fractalkine Binding Site in the CX3CR1 Isoforms

**Figure 2:**
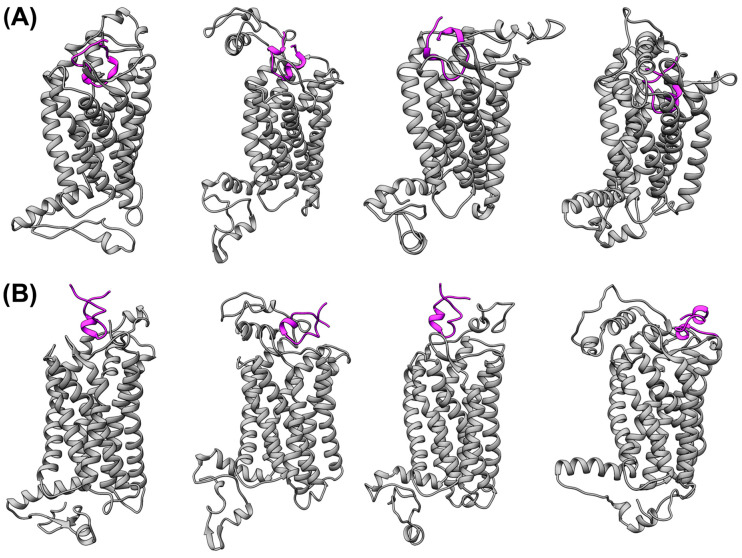
Molecular docking of cysteine-noose domain of hRSV G protein (cndG) with the CX3CR1 isoforms. The structural models of the complexes of the transmembrane receptor CX3CR1 with cndG calculated by ClusPro (**A**) and HADDOCK (**B**) servers are denoted for isoforms 1 to 4 from left to right, respectively. The CX3CR1 isoforms are represented as gray cartoons, while cndG is denoted as a purple cartoon.

The molecular docking calculations were performed using the representative structures obtained from the clustering analysis performed for 300 ns simulations of the structural models of the CX3CR1 isoforms as determined by the RoseTTAFold server. For these calculations, different servers were used, as mentioned in Section 2.2. Among these servers, ClusPro and HADDOCK showed satisfactory results, positioning the α-helices of cndG directed towards the putative binding site in the N-terminal cavity of the CX3CR1 isoforms (Figure 2). The search for this guidance among the results of different servers was based on the interaction of fractalkine (CX3CL1) with receptors CX3CR1 isoform 1 and US28 (Figure S6) [30,31]. Comparing the molecular docking results in Figure 2, it is possible to notice that, for the ClusPro structural models of the complex, cndG is more buried in the putative binding site in the cavity of the N-terminal portion of the barrel of α-helices of the CX3CR1 isoforms, while for the HADDOCK models, it is located on the entrance of the binding cavity. We proceed with 300 ns MD simulations in the POPC lipid bilayer for all the structural models of the complex determined by ClusPro and HADDOCK since there is no experimental structural information regarding the CX3CR1/cndG interaction and thus the conformational diversity shown by the two servers can contribute significantly to characterizing this interaction.

**Figure 3:**
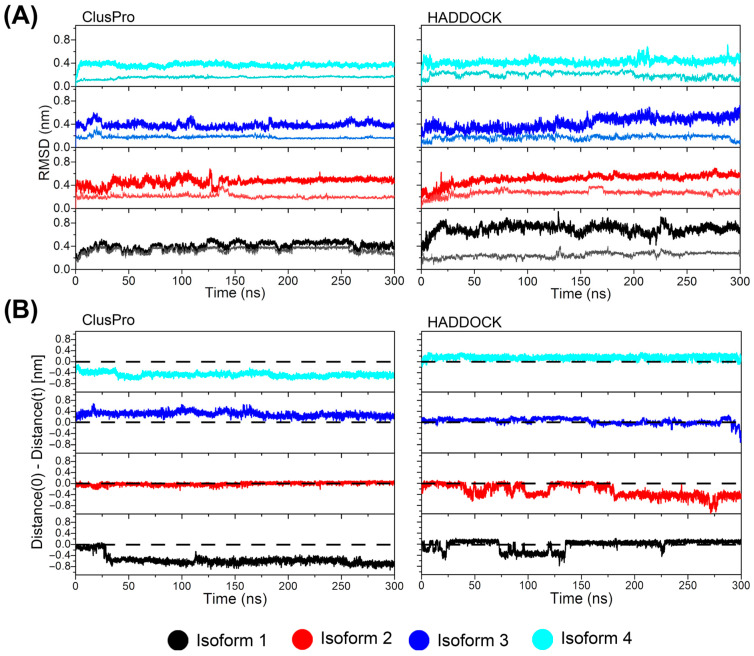
Structural stability of the models of the CX3CR1/cndG complexes throughout the MD trajectories. (**A**) RMSD values were calculated using two different structural alignments: the thick line denotes the RMSD of the cndG concerning the alignment for the CX3CR1 backbone atoms, while the thin line represents the RMSD of the cndG concerning the structural alignment for itself. (**B**) Relative distances between the center of mass of the cndG and CX3CR1 isoforms. Distance (0) means the distance from the initial conformation of the structural model of the complexes comes from the docking calculations, while distance (t) represents the distances from subsequent frames taken from the MD trajectories. Positive values indicate moving apart from each other, while negative values mean that they approach. The results for the isoforms 1 to 4 are presented in black, red, blue, and cyan colors, respectively.

The RMSD values calculated for the cndG backbone along the 300 ns MD trajectory, when the structural alignment is performed on its backbone (Figure 3A, thin line), reveal stability plateaus around 2 to 3 Å for all structural models of the complexes (ClusPro and HADDOCK), suggesting that cndG does not undergo significant conformational changes throughout the simulations. For the alignment performed taking the CX3CR1 backbone as a reference (Figure 3A, thick line), RMSD values for the cndG backbone are observed to reach stability around 4 to 6 Å for all complex models, indicating that cndG may have undergone slight variations in its positioning concerning the initial position (docking model) in the putative binding site in the cavity of CX3CR1 isoforms, either moving closer and/or moving away. To determine how much the position of cndG changes concerning its starting point (docking model) over 300 ns of MD simulation, we calculated the distance between the centers of mass of the cndG and of the barrel of α-helices of the CX3CR1 isoforms and cndG, taking the starting point of the simulation (t = 0 ns) as a reference point. From Figure 3B, which shows distance difference values concerning the reference point at t = 0 ns, it is possible to observe that cndG remains in the putative binding site of the N-terminal cavity of the CX3CR1 isoforms throughout the simulation for all structural models of the complexes determined by ClusPro and HADDOCK, since the differences in distances reach a maximum value in modulus of 4 Å, sometimes positive when CX3CR1 and cndG move slightly apart and sometimes negative when they approach each other. This result corroborates the analysis of the number of contacts < 0.6 nm between CX3CR1 isoform and cndG that remains stable throughout the simulations (Figure S7), and the representative structures of the complexes obtained from clustering analyses of their 300 ns MD trajectories since the cndG position does not present significant changes concerning the initial position of the docking structural models (Figure S8). This suggests that the interaction of the cndG with the canonical fractalkine binding site in the N-terminal cavity of the CX3CR1 isoforms is structurally stable. It is worth mentioning that the RMSD values for the backbone atoms of the transmembrane receptor show that no CX3CR1 isoforms presented significant conformational changes over the 300 ns of simulations for the structural models of the complexes determined by ClusPro and HADDOCK (Figure S9).

### 3.3. Identification of Key Residues for Stabilization of the CX3CR1/cndG Interaction from Hydrogen Bonds and MM-GBSA Analysis

**Figure 4:**
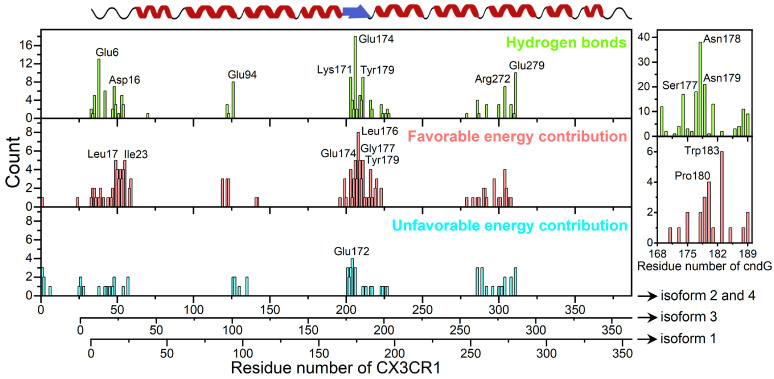
Key residues in the interactions of cndG with CX3CR1 isoforms. Count (frequency of occurrence) of the amino acid residues involved in hydrogen bonds (green) with persistency > 10% and favorable (red) and unfavorable (cyan) energy contributions to $∆G_{b}$ for the CX3CR1/cndG interaction. On the left, the amino acid residues of the transmembrane receptor are represented, while on the right the counts for the cndG residues are shown. The highlighted residues are those that appeared most. The secondary structure of transmembrane receptor CX3CR1 is denoted on the top.

**Figure 5:**
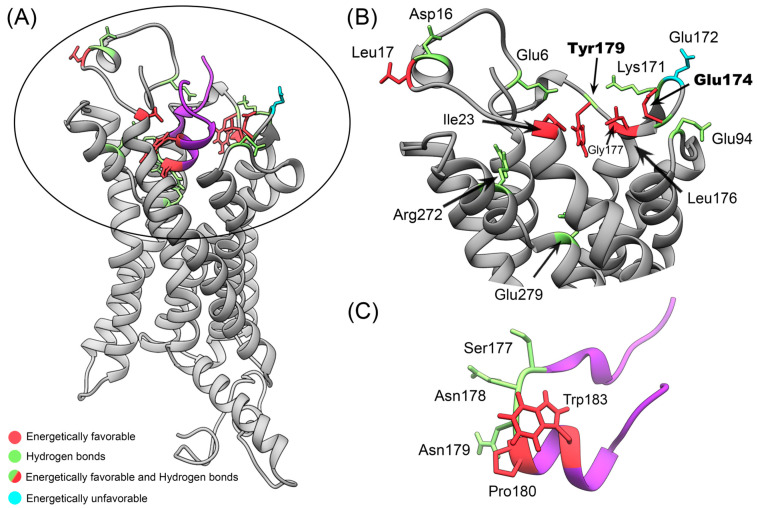
Structural detail of the canonical fractalkine binding site of the transmembrane receptor CX3CR1 for interacting with cndG. (**A**) Structural model of the complex formed between CX3CR1 (gray) and cndG (purple), showing the key residues identified from the analysis of hydrogen bonds with persistency >10% and energy contributions to $∆G_{b}$. The residues showing a significant count of hydrogen bonds with persistency >10% and favorable and unfavorable energy contributions are highlighted in green, red, and cyan colors, respectively. (**B**) Zoom of the canonical fractalkine binding site region circulated in (**A**), emphasizing the key residues with a focus on Tyr179 and Glu174, which stand out in both the analysis of hydrogen bonds and favorable energy contribution. (**C**) Representation of the cndG with the key residues for hydrogen bonds and favorable energy contribution to $∆G_{b}$ highlighted in green and red, respectively.

We perform the calculations of the number of hydrogen and theoretical Gibbs free energy of binding ($∆G_{b}$) using MM-GBSA to identify and characterize CX3CR1 and cndG amino acid residues responsible for the structural stabilization of the docking models of the complexes determined by ClusPro and HADDOCK. Using broad conformational space from eight different 300 ns MD trajectories of the structural models of the CX3CR1/cndG complexes, we looked for residues common to all isoforms that are involved in pivotal interactions. The evaluation of the hydrogen bonds formed between CX3CR1 isoforms and cndG reveals that this non-covalent interaction represents an important aspect in the structural stability of the models of all complexes since the number of hydrogen bonds remains stable over the 300 ns MD simulation (Figure S10). Using the number of hydrogen bonds along the trajectories of the structural models of the complexes, we also calculate the percentages of persistence of these bonds, and those with persistence values higher than 10% (Tables S2 and S3) were counted concerning the amino acid residues involved in, either as a donor or acceptor of a hydrogen bond, both CX3CR1 isoforms and cndG (Figure 4). Using CX3CR1 isoform 1 residue numbering as a reference, noting that isoforms 2 and 4 have 32 more residues in the N-terminal and isoform 3 only 7, it is possible to observe from Figure 4 that Glu6, Asp16, Glu94, Lys171, Glu174, Tyr179, Arg272, and Glu279 are the residues of CX3CR1 isoforms more often involved in hydrogen bonds along all MD simulations. These residues comprehensively map the binding cavity in the N-terminal portion of the CX3CR1 cell receptor (Figure 5A,B). It is worth mentioning that the residue Glu174, which shows the highest number of counts (Figure 4A), is on the β-strand of the ECL2 region that directs the docking calculations on the canonical fractalkine binding site in the N-terminal cavity of the CX3CR1 isoforms (Figure 5). Regarding cndG, the residues Ser177, Asn178, and Asn179 often participate in hydrogen bonds with CX3CR1 isoforms (Figure 4) and thus are fundamental for the interaction with the cell receptor (Figure 5A,C).

From the MM-GBSA calculations, we identified that the residues of CX3CR1 isoforms and cndG contribute favorably and unfavorably to the binding free energy for the structural models of the complexes determined by ClusPro and HADDOCK (Figures S11 and S12). The favorable and unfavorable energy contributions to $∆G_{b}$ of the residues with values higher than the average plus standard deviation for each isoform and cndG were counted (Tables S4–S7) and shown in Figure 4, respectively. As for the hydrogen analysis, we used CX3CR1 isoform 1 residue numbering as a reference to describe the fundamental contribution to $∆G_{b}$. It is possible to observe from Figure 4 that the residues Leu17, Ile23, Glu174, Leu176, Gly177, and Tyr179 of CX3CR1 isoforms stand out for often participating in favorable energy contribution over all MD simulations. Unlike the favorable contributions to $∆G_{b}$, unfavorable energy contributions were more dispersed among the residues, not presenting a significant count among all isoforms, except for Glu172 (Figure 4). It is noteworthy that Glu174, Leu176 (highest number of counts), Gly177, and Tyr179 are residues located in the ECL2 region used to drive the docking calculations, highlighting the favorable contribution of this region to the structural models of the CX3CR1/cndG complexes. Note that Glu174 and Tyr179 are also residues often involved in hydrogen bonds with cndG, which reinforces their importance to the canonical fractalkine binding site in the N-terminal cavity of the CX3CR1 isoforms. As well as hydrogen-bonded residues of the isoforms, the significative energy contribution to the $∆G_{b}$ of these residues is also comprehensively mapped to the binding cavity in the N-terminal portion of the transmembrane receptor CX3CR1 (Figure 5A,B). Concerning cndG, Pro180 and Trp183 are residues often involved in favorable energy contribution in the interaction with isoforms (Figure 4) and therefore are important for stabilizing the binding to the cell receptor CX3CR1 (Figure 5A,C).

A combined analysis of hydrogen bonds with persistence >10% and favorable energy contributions to $∆G_{b}$ for a broad conformational space coming from eight different 300 ns MD simulations of structural models of the CX3CR1/cndG complexes provided the identification of key residues for this interaction, which corroborate the cryo-EM and crystal structures determined for the complexes formed between the chemokine fractalkine and the receptors CX3CR1 and US28 [31,32]. It is noteworthy that we did not observe significant contributions of the N-terminal extensions of CX3CR1 isoforms to the interaction with cndG that could indicate a potential binding preference. Burg and collaborators evaluated the interaction of CX3CL1 with the transmembrane receptor US28 using hydrogen bonds and van der Waals contacts [32]. They identified Glu18 and Phe25 at the N-terminal region and the residues Lys169, Gln172, Met174, Thr175, Tyr177, Lys270, and Glu277 in the ECL2 region of the receptor US28 [32], which correspond, respectively, to the residues Asp16, Ile23, Lys171, Glu174, Leu176, Gly177, Tyr179, Arg272, and Glu279 of CX3CR1 (numbering referring to isoform 1) observed in the present study based on the sequence and structure alignment (Figure S13). These residues are also conserved in homologous CX3CR1 sequences from other species (Figure S14). It is noteworthy that Gln172 and Glu277 of US28 were reported by Burg and coauthors as the residues with the largest molecular contact networks [32], and the interactions performed by Glu277 provide a structural basis for the observation that this residue is important for chemokine receptor signaling [60]. We identified the corresponding residues Glu174 and Glu279 of CX3CR1, also with molecular contact networks fundamental for the interaction with cndG. Due to the lack of resolution of the side chains of the ECL2 region of the CX3CR1/Gi1 complex in the CX3CL1-bound states for the cryo-EM structure, Lu and collaborators did not report the existing non-covalent interactions of this region with fractalkine but showed the participation of the residue Glu279 of the transmembrane receptor in polar contacts with this chemokine [31]. Lu and coauthors also showed that mutating Glu279 to alanine completely abolishes the signaling of CX3CR1, indicating the key role of this residue in the molecular recognition of CX3CL1 [31] and once again corroborating its importance in the interaction with the cysteine-noose domain of the hRSV G protein.

## 4. Conclusions

The results presented here provide, for the first time from a structural point of view, a comprehensive molecular characterization of the interaction of the cysteine-noose domain of hRSV G protein (cndG) with the transmembrane receptor CX3CR1 isoforms thanks to the use of computational tools such as modeling, docking, and molecular dynamics simulations. This interaction is fundamental for the attachment process of the hRSV with the host cell in viral cycle replication. The interaction of the cndG with the canonical fractalkine binding site in the N-terminal cavity of the CX3CR1 isoforms revealed structural stability along 300 ns trajectories for eight different structural models of these complexes, totalizing 2.4 μs MD simulations in a POPC lipid bilayer that characterize a broad conformational space for the studied system. From the analysis of these trajectories, we identified that residues in the ECL2 region and Glu279 of the CX3CR1 isoforms are fundamental for stabilizing the cndG interaction in the canonical fractalkine binding site, which corroborates with cryo-EM and crystal structure data for the binding of CX3CL1 to transmembrane receptors CX3CR1 and US28 [31,32]. These findings obtained in the present study contribute to the understanding of the interaction between the transmembrane receptor CX3CR1 isoforms and the conserved cysteine-noose domain of hRSV G protein, helping us to elucidate molecular details in the virus-cell attachment process and paving new paths in the search for potential virucidal candidates that could act as inhibitors of the CX3CR1/G interaction. It is worth mentioning that since the chemokine fractalkine shares structural similarities with cndG, the design of mimetic peptides of this G domain could be considered as a possible strategy for the development of inhibitors not only of the CX3CR1/G interaction but also of the binding of CX3C chemokines to this transmembrane receptor. As the promiscuity property of chemokines and cognate chemokine receptors causes difficulty in drug development, CX3CR1 shows a unique advantage as a potential drug target [31]. Nevertheless, the drug development targeting CX3CR1 is relatively slow and challenging [61,62,63]. Until now, only one high-affinity small-molecule inhibitor, AZD8797, and an anti-CX3CR1 nanobody are in phase 1 clinical trials to treat cancer pain and kidney disease, respectively [64,65]. In a broader context, the present study contributes as a molecular basis, guiding, for instance, the development of novel compounds targeting the CX3CR1/CX3C-motif signaling axis activity.

## Data Availability

The original contributions presented in the study are included in the article/Supplementary Material, further inquiries can be directed to the corresponding author/s.

## Supplementary Materials

The following supporting information can be downloaded at https://www.mdpi.com/article/10.3390/membranes14040084/s1, Figure S1: Alignment of amino acid sequences for the isoforms of the cellular receptor CX3CR1 conducted using the Clustal Omega server; Figure S2: Structural alignment of CX3CR1 from cryo-EM with models from servers RoseTTAFold and trRosetta; Figure S3: RMSD and number of contacts for the MD simulations of the structural models of isoform 2, 3 and 4; Figure S4: Representative structures of CX3CR1 isoforms 2, 3, and 4 in POPC lipid bilayer; Figure S5: Mass density profile for the POPC lipid bilayer for the four MD simulations with the CX3CR1 isoforms; Figure S6: Tridimensional structure of the US28/fractalkine and CX3CR1/fractalkine complexes; Figure S7: Number of contacts between CX3CR1 isoforms and cndG in the complexes from ClusPro and HADDOCK; Figure S8: Comparison between the positions of the cndG in the structural models of the molecular docking calculations and in the representative structures of the complexes from MD simulations; Figure S9: RMSD values of the CX3CR1 isoforms and cndG along the MD simulations; Figure S10: Number of CX3CR1/cndG hydrogen bonds along the MD simulations; Figure S11: Energy contributions of the residues of CX3CR1 isoforms and cndG to total binding free energy of the complexes for the structural models from server ClusPro; Figure S12: Energy contributions of the residues of CX3CR1 isoforms and cndG to total binding free energy of the complexes for the structural models from server HADDOCK; Figure S13: Sequence alignment of CX3CR1 and US28 receptors and comparison of its residues in the binding cavity; Figure S14: Multiple sequence alignment for homologous CX3CR1s of different species; Table S1: Values of the percentages of the ψ and ϕ torsional angles of the residues of the structural models of the CX3CR1 isoform 1 determined from Ramachandran plot; Table S2: Percentage of persistence of hydrogen bonds > 10% for the structural models of the complexes of cndG with CX3CR1 isoforms 1, 2, 3, and 4 from ClusPro server; Table S3: Percentage of persistence of hydrogen bonds > 10% for the structural models of the complexes of cndG with CX3CR1 isoforms 1, 2, 3, and 4 from HADDOCK server; Table S4: The most significant contributions of binding free energy via MM-GBSA of the residues of isoform 1, 2, 3, and 4 for the structural models of the CX3CR1/cndG complexes from the ClusPro server; Table S5: The most significant contributions of binding free energy via MM-GBSA of the residues of isoform 1, 2, 3, and 4 for the structural models of the CX3CR1/cndG complexes from the HADDOCK server; Table S6: The most significant contributions of binding free energy via MM-GBSA of the residues of cndG for the four structural models of the CX3CR1/cndG complexes from the ClusPro server; Table S7: The most significant contributions of binding free energy via MM-GBSA of the residues of cndG for the four structural models of the CX3CR1/cndG complexes from the HADDOCK server.
