# Supplementary material for: Computational Insights into the Interaction of the Conserved Cysteine-Noose Domain of the Human Respiratory Syncytial Virus G Protein with the Canonical Fractalkine Binding site of Transmembrane Receptor CX3CR1 Isoforms
Source: Membranes (Basel). 2024 Apr 4;14(4):84. doi: 10.3390/membranes14040084 (PMC11052111; doi:10.3390/membranes14040084)
Supplement: Supplementary file 1 [file membranes-14-00084-s001.zip › membranes-2924508-supplementary.pdf]

Computational Insights into the Interaction of the Conserved Cysteine-Noose Domain of the Human Respiratory Syncytial Virus G Protein with the Canonical Fractalkine Binding Site of Transmembrane Receptor CX3CR1 Isoforms

João Victor Piloto[1,†]; Raphael Vinicius Rodrigues Dias[1,†]; Wan Suk Augusto Mazucato[1], Marcelo Andres Fossey[1], Fernando Alves de Melo[1], Fabio Ceneviva Lacerda Almeida[2], Fatima Pereira de Souza[1], Icaro Putinhon Caruso[1,*]

[1]Multiuser Center for Biomolecular Innovation (CMIB), Department of Physics, São Paulo State University (UNESP), São Jose do Rio Preto 15054-000, Brazil.

[2]Institute of Medical Biochemistry (IBqM), National Center of Nuclear Magnetic Resonance Jiri Jonas, Federal University of Rio de Janeiro, Rio de Janeiro 21941-902, Brazil.

† The authors contributed equally to this work.

***Address correspondence to:** Icaro Putinhon Caruso, São Paulo State University "Júlio de Mesquita Filho" - UNESP, Institute of Biosciences, Humanities and Exact Sciences - IBILCE, Department of Physics, Rua Cristóvão Colombo, 2265, CEP 15054-000, São José do Rio Preto, São Paulo, Brazil, Tel.: +55-17-3221-2515, e-mail: icaro.caruso@unesp.br

SUPPLEMENTARY MATERIAL

Figure S1. Alignment of amino acid sequences for the isoforms of the cellular receptor CX3CR1 conducted using the Clustal Omega server. The primary sequence of isoform 1 is represented in black, isoform 2 in red, isoform 3 in blue, and isoform 4 in green. It is noteworthy that isoforms 2 and 4 exhibit 32 additional residues compared to isoform 1, while isoform 3 contains 7 extra residues relative to isoform 1.

```
Isoform 1       --------------------------------MDQFPESVTENFEYDDLAEACYIGDIVV
Isoform 2       MREPLEALKLADLDFRKSSLASGWRMASGAFTMDQFPESVTENFEYDDLAEACYIGDIVV
Isoform 3       -------------------------MASGAFTMDQFPESVTENFEYDDLAEACYIGDIVV
Isoform 4       MREPLEAFKLADLDFRKSSLASGWRMASGAFTMDQFPESVTENFEYDDLAEACYIGDIVV
                                                ****************************

Isoform 1       FGTVFLSIFYSVIFAIGLVGNLLVVFALTNSKKPKSVTDIYLLNLALSDLLFVATLPFWT
Isoform 2       FGTVFLSIFYSVIFAIGLVGNLLVVFALTNSKKPKSVTDIYLLNLALSDLLFVATLPFWT
Isoform 3       FGTVFLSIFYSVIFAIGLVGNLLVVFALTNSKKPKSVTDIYLLNLALSDLLFVATLPFWT
Isoform 4       FGTVFLSIFYSVIFAIGLVGNLLVVFALTNSKKPKSVTDIYLLNLALSDLLFVATLPFWT
                ************************************************************

Isoform 1       HYLINEKGLHNAMCKFTTAFFFIGFFGSIFFITVISIDRYLAIVLAANSMNNRTVQHGVT
Isoform 2       HYLINEKGLHNAMCKFTTAFFFIGFFGSIFFITVISIDRYLAIVLAANSMNNRTVQHGVT
Isoform 3       HYLINEKGLHNAMCKFTTAFFFIGFFGSIFFITVISIDRYLAIVLAANSMNNRTVQHGVT
Isoform 4       HYLINEKGLHNAMCKFTTAFFFIGFFGSIFFITVISIDRYLAIVLAANSMNNRTVQHGVT
                ************************************************************

Isoform 1       ISLGVWAAAILVAAPQFMFTKQKENECLGDYPEVLQEIWPVLRNVETNFLGFLLPLLIMS
Isoform 2       ISLGVWAAAILVAAPQFMFTKQKENECLGDYPEVLQEIWPVLRNVETNFLGFLLPLLIMS
Isoform 3       ISLGVWAAAILVAAPQFMFTKQKENECLGDYPEVLQEIWPVLRNVETNFLGFLLPLLIMS
Isoform 4       ISLGVWAAAILVAAPQFMFTKQKENECLGDYPEVLQEIWPVLRNVETNFLGFLLPLLIMS
                ************************************************************

Isoform 1       YCYFRIIQTLFSCKNHKKAKAIKLILLVVIVFFLFWTPYNVMIFLETLKLYDFFPSCDMR
Isoform 2       YCYFRIIQTLFSCKNHKKAKAIKLILLVVIVFFLFWTPYNVMIFLETLKLYDFFPSCDMR
Isoform 3       YCYFRIIQTLFSCKNHKKAKAIKLILLVVIVFFLFWTPYNVMIFLETLKLYDFFPSCDMR
Isoform 4       YCYFRIIQTLFSCKNHKKAKAIKLILLVVIVFFLFWTPYNVMIFLETLKLYDFFPSCDMR
                ************************************************************

Isoform 1       KDLRLALSVTETVAFSHCCLNPLIYAFAGEKFRRYLYHLYGKCLAVLCGRSVHVDFSSSE
Isoform 2       KDLRLALSVTETVAFSHCCLNPLIYAFAGEKFRRYLYHLYGKCLAVLCGRSVHVDFSSSE
Isoform 3       KDLRLALSVTETVAFSHCCLNPLIYAFAGEKFRRYLYHLYGKCLAVLCGRSVHVDFSSSE
Isoform 4       KDLRLALSVTETVAFSHCCLNPLIYAFAGEKFRRYLYHLYGKCLAVLCGRSVHVDFSSSE
                ************************************************************

Isoform 1       SQRSRHGSVLSSNFTYHTSDGDALLLL
Isoform 2       SQRSRHGSVLSSNFTYHTSDGDALLLL
Isoform 3       SQRSRHGSVLSSNFTYHTSDGDALLLL
Isoform 4       SQRSRHGSVLSSNFTYHTSDGDALLLL
                ***************************
```

Figure S2. Structural alignment of CX3CR1 from cryo-EM with models from servers RoseTTAFold and trRosetta. Structure of the CX3CR1 receptor resolved by cryo-EM (PBD 7XBX) colored in gray. The structures calculated via molecular modeling via RoseTTAFold and trRosetta are superimposed, with the RMSD values highlighted via a color gradient, going from blue to red.

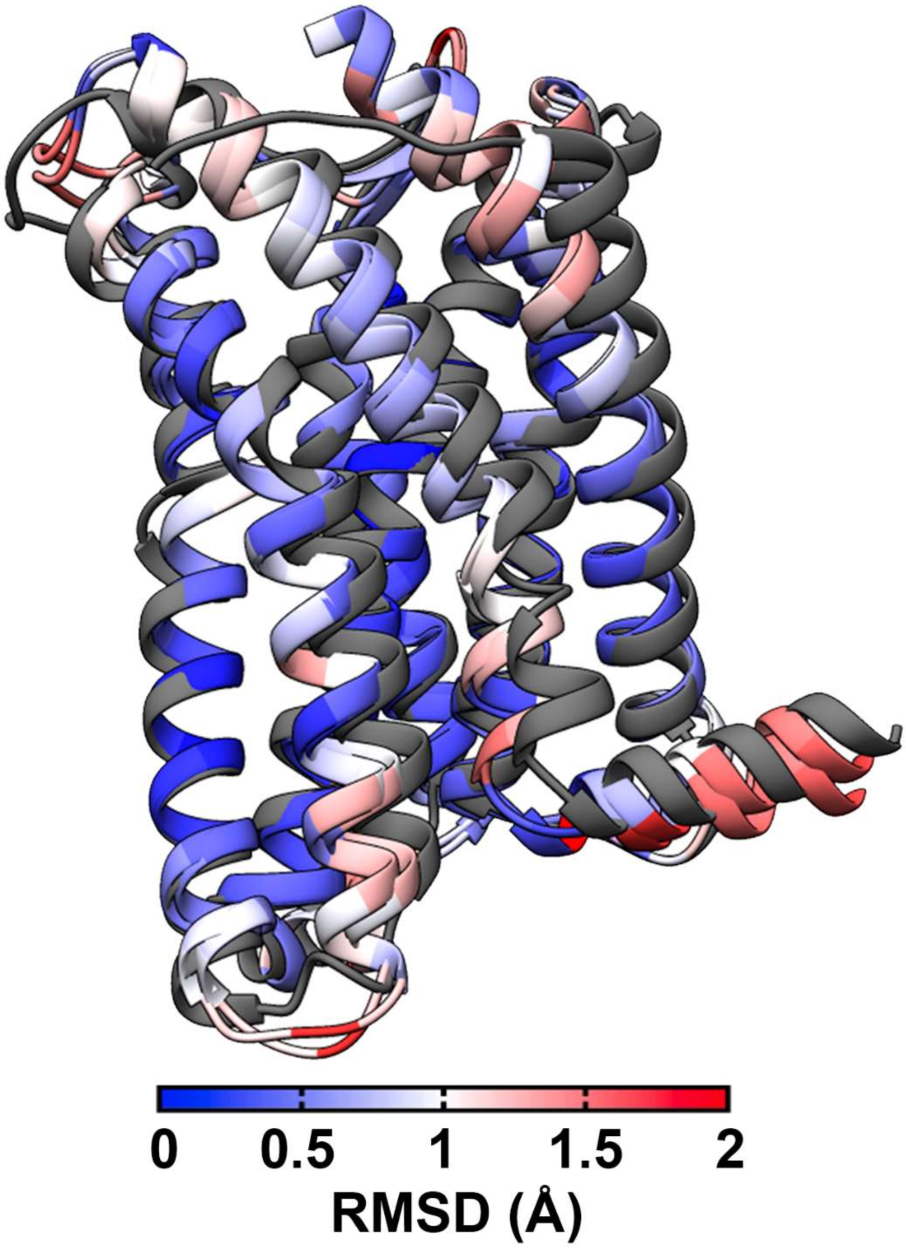

Figure S3. RMSD and number of contacts for the MD simulations of the structural models of isoform 2, 3 and 4. RMSD analysis of the main chain atoms (A) and the number of contacts < 0.6 nm between the N-terminal residues and the β-strand Glu174–Gly177 in the ECL2 region (B) along the 300 ns MD trajectories for the structural models of isoforms 2 (black), 3 (red), and 4 (blue) determined by the RoseTTAFold servers.

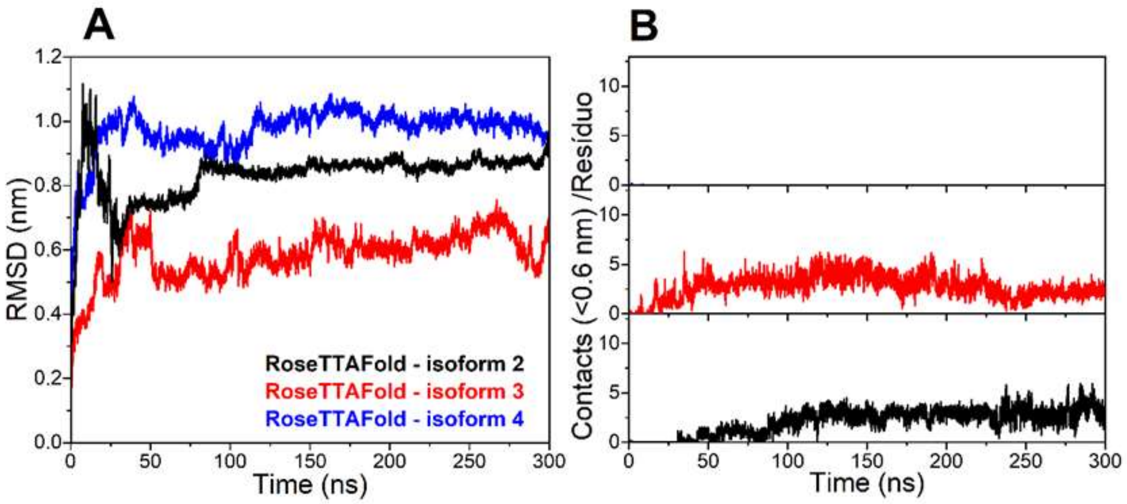

Figure S4. Representative structures of CX3CR1 isoforms 2, 3, and 4 in POPC lipid bilayer. The representative structures for isoform 2 (A), isoform 3 (B), and isoform 4 (C) in a POPC lipid bilayer were obtained through cluster analysis of the 300 ns MD simulations. The protein is depicted in a cartoon model, and lipids are represented as sphere and line models. The barrel of α-helices of the protein is denoted in gray, the N-terminal region in yellow, and the C-terminal region in orange. The phosphorus atom of the lipid polar head is shown as a blue sphere, and the hydrophobic tail as gray lines.

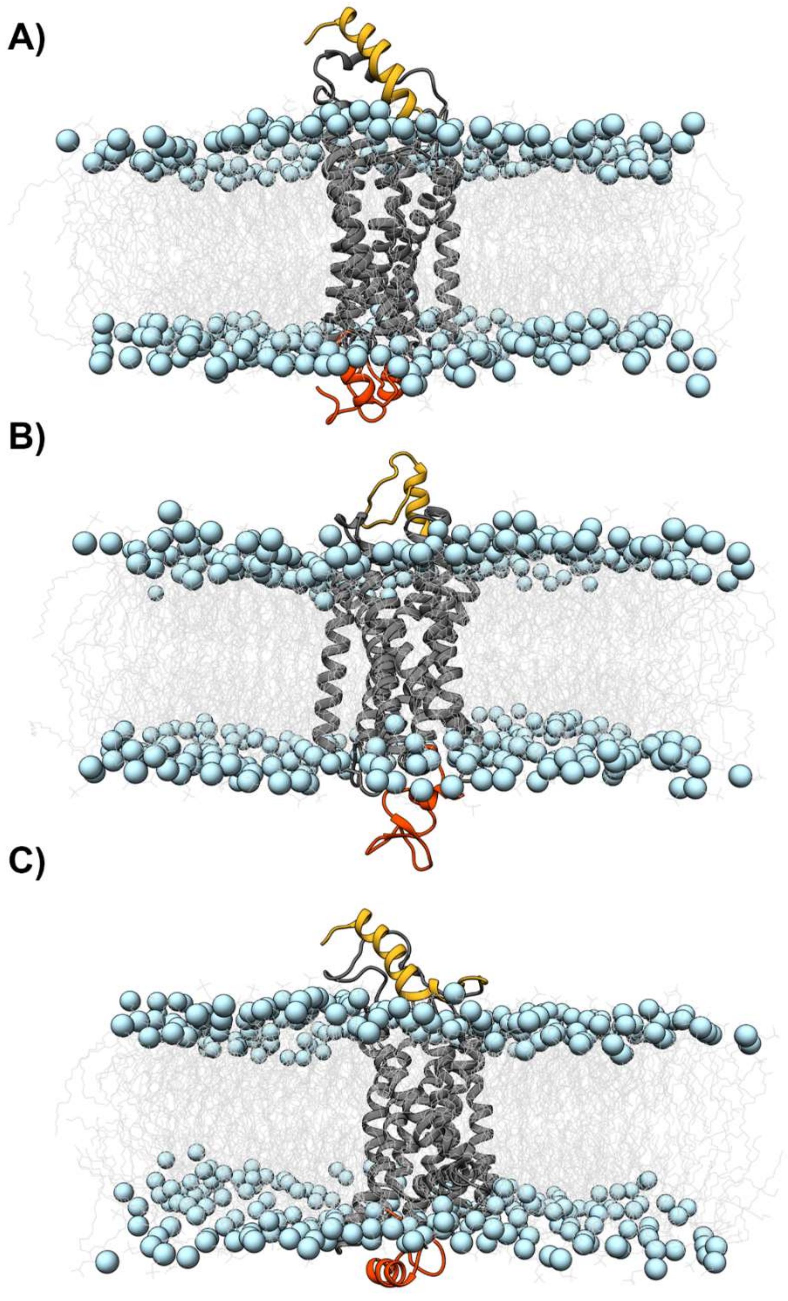

Figure S5. Mass density profile for the POPC lipid bilayer for the four MD simulations with the CX3CR1 isoforms. The profile for the simulations with the isoform 1 is presented in (A), 2 in (B), 3 in (C), and 4 in (D). The bilayer thickness is determined by the P8-P8 distance. The apolar part of the POPC bilayer is indicated by a black line, P8 phosphorus atoms with a red line, the protein with a green line, and water molecules with a blue line.

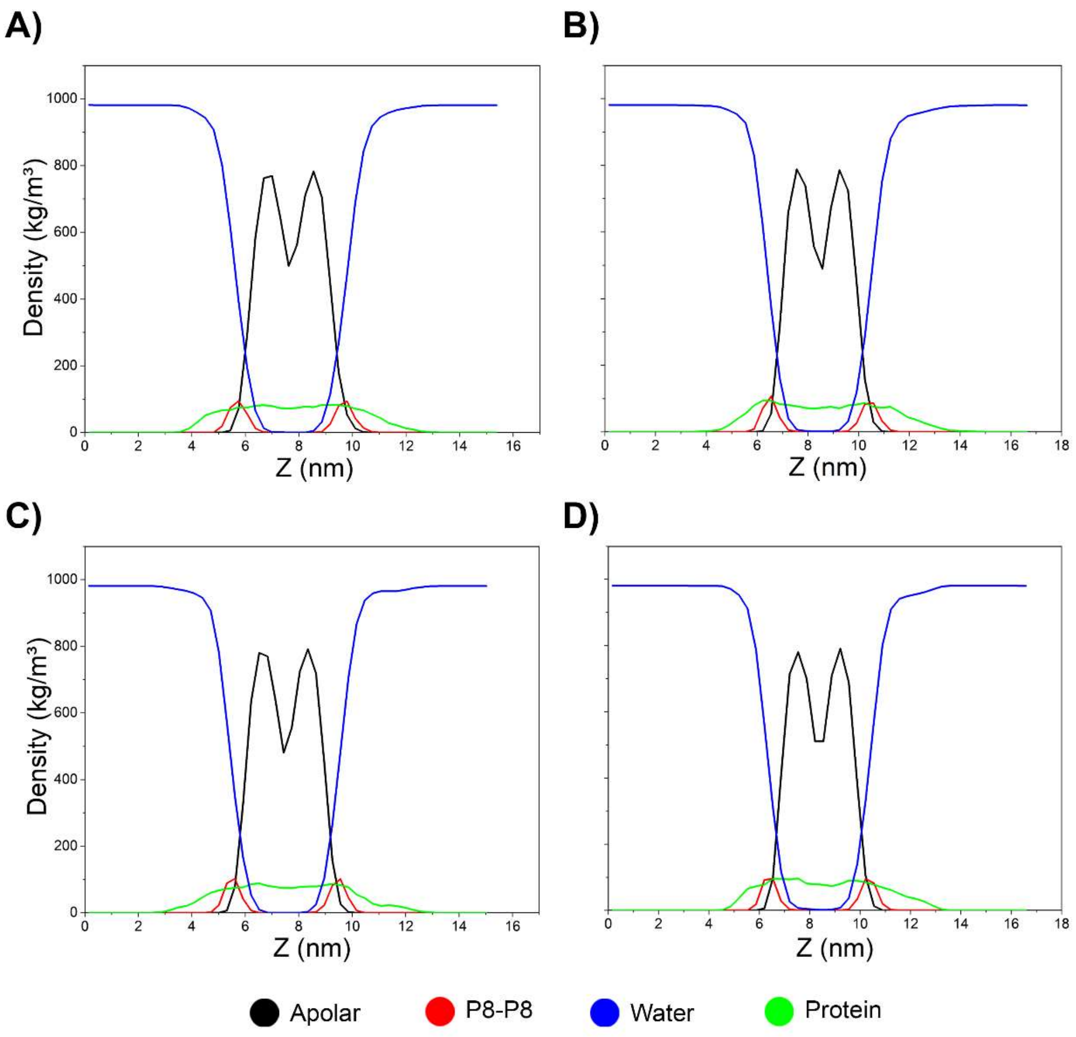

Figure S6. Tridimensional structure of the US28/fractalkine and CX3CR1/fractalkine complexes. (Left) Structure (PDB 5WB2) of the complex formed between fractalkine CX3CL1 (magenta) and the homologous receptor US28 (gray). (Right) Structure of the CX3CR1 receptor resolved by cryo-EM (PDB 7XBX) anchored with the fractalkine (CX3CL1). The proteins are represented using the cartoon model. The corresponding β-strands in the ECL2 region of the CX3CR1 homolog (Glu174–Gly177) is highlighted in cyan in the protein's binding cavity.

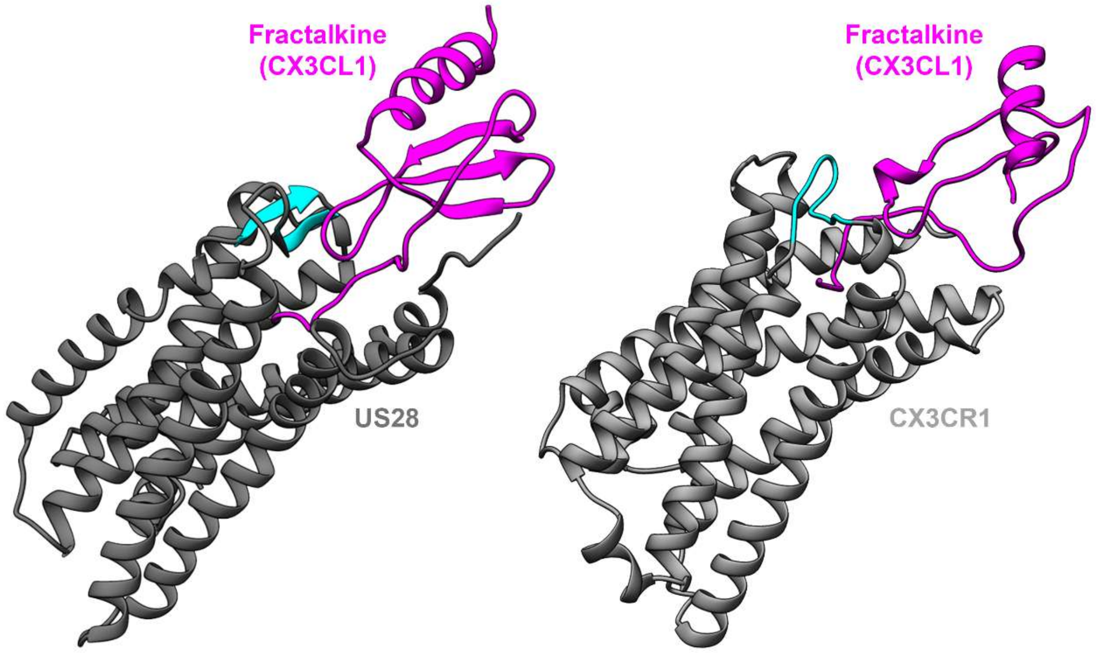

Figure S7. Number of contacts between CX3CR1 isoforms and cndG in the complexes from ClusPro and HADDOCK. The number of contacts < 0.6 nm determined between the atoms of the isoforms and cndG over the 300 ns simulation for the structural models of the complexes calculated by ClusPro (left) and HADDOCK (right). The results for each isoform are presented in the following color scheme: black for isoform 1, red for 2, blue for 3, and cyan for 4.

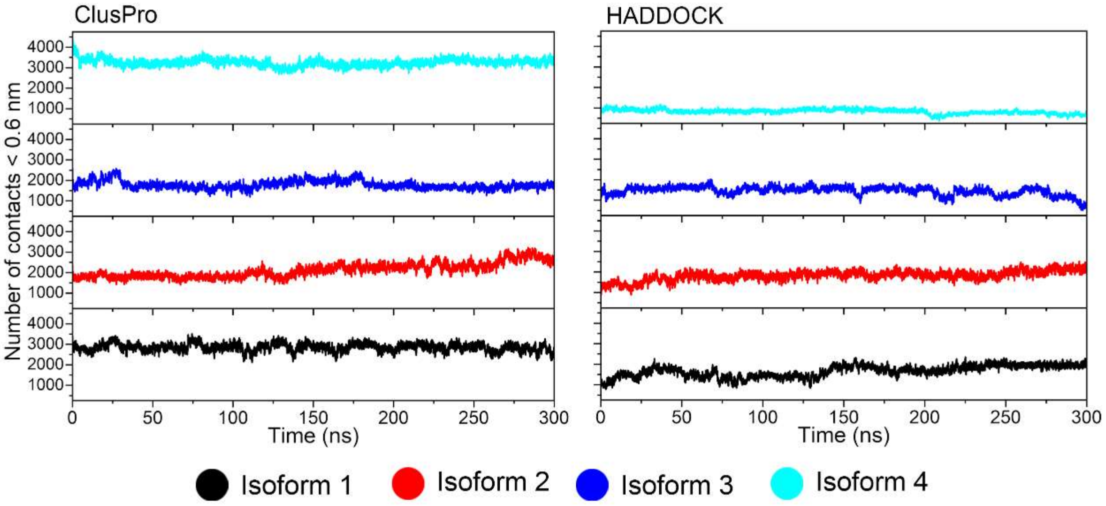

Figure S8. Comparison between the positions of the cndG in the structural models of the molecular docking calculations and in the representative structures of the complexes from MD simulations. The structural models of the CX3CR1/cndG complexes from ClusPro and HADDOCK are denoted in (A) and (B), respectively. The protein is presented as a cartoon model. The cndG is shown as a cartoon in magenta for the docking model and in yellow for the representative structure from clustering analysis.

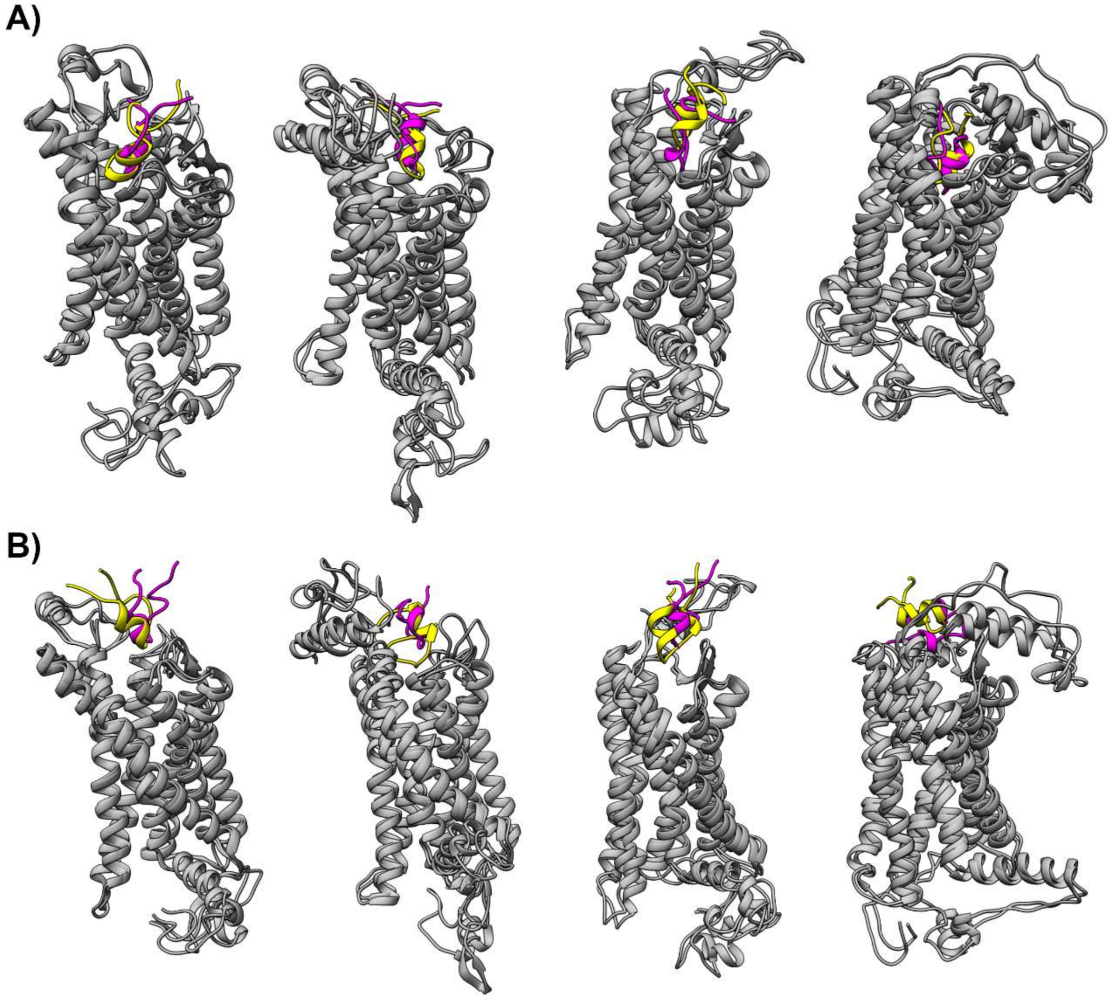

Figure S9. RMSD values of the CX3CR1 isoforms and cndG along the MD simulations. The values of RMSD calculated for the main chain atoms of the isoforms and the cndG over the 300 ns MD simulation for the structural models of the complexes determined by the servers ClusPro and HADDOCK. The results for each isoform are presented in the following color scheme: black for isoform 1, red for 2, blue for 3, and cyan for 4.

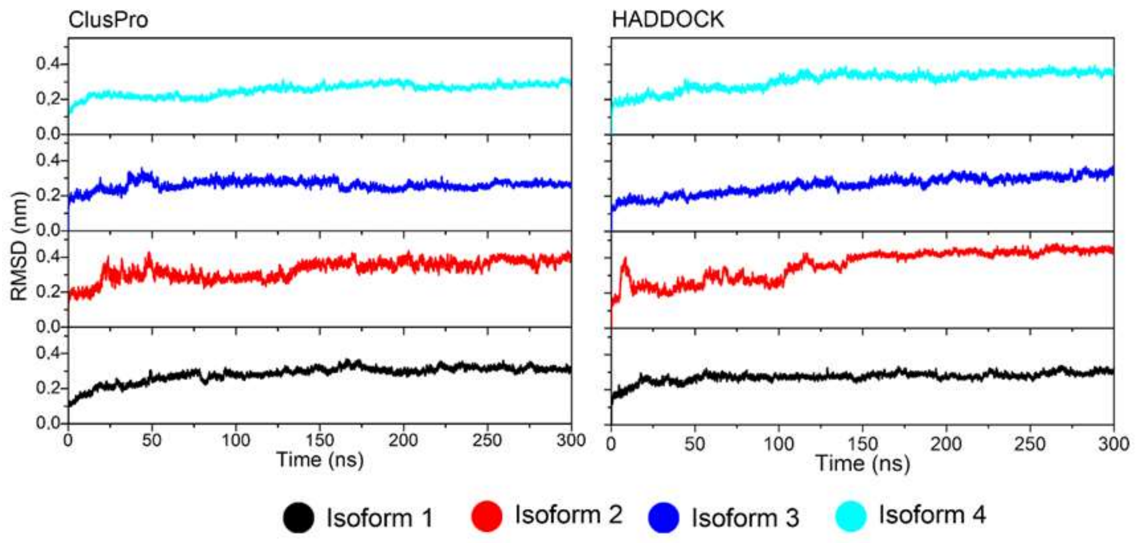

Figure S10. Number of CX3CR1/cndG hydrogen bonds along the MD simulations. The values of number of hydrogen bonds calculated between atoms of the isoforms and the cndG over the 300 ns MD simulation for the structural models of the complexes determined by the servers ClusPro and HADDOCK. The results for each isoform are presented in the following color scheme: black for isoform 1, red for 2, blue for 3, and cyan for 4.

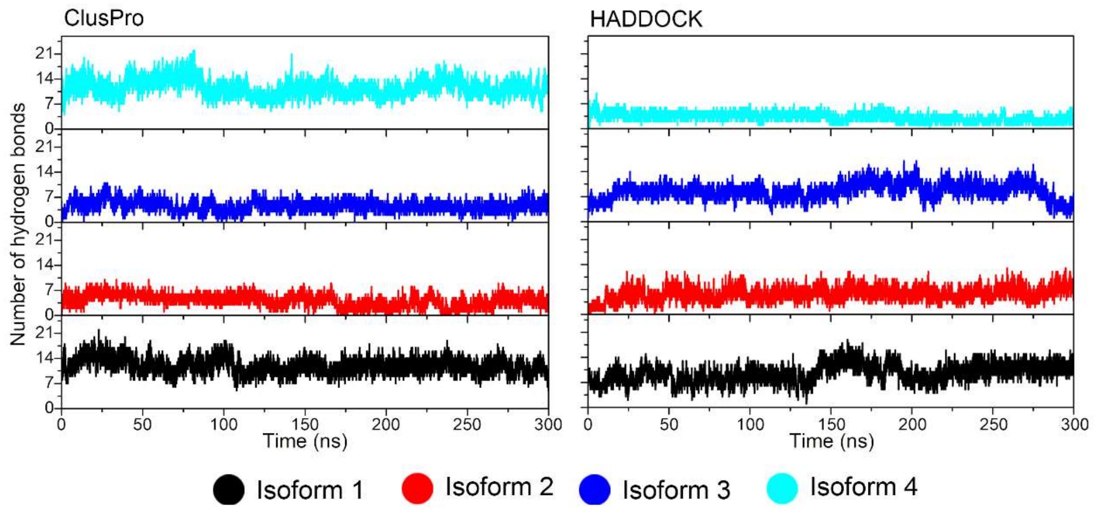

Figure S11. Energy contributions of the residues of CX3CR1 isoforms and cndG to total binding free energy of the complexes for the structural models from server ClusPro. Favorable (red) and unfavorable (blue) contributions of the amino acid residues of the CX3CR1 isoforms (A) and cndG (B) in the four complexes calculated via MM-GBSA along the MD trajectories.

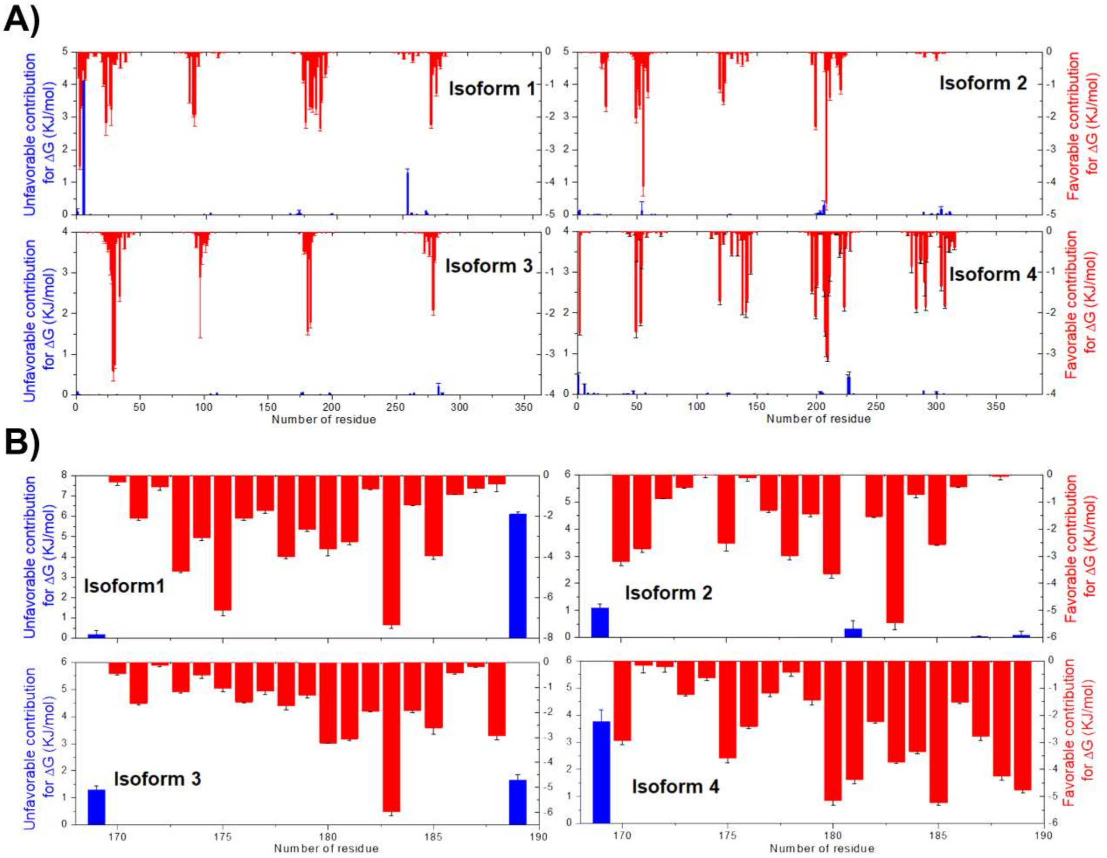

Figure S12. Energy contributions of the residues of CX3CR1 isoforms and cndG to total binding free energy of the complexes for the structural models from server HADDOCK. Favorable (red) and unfavorable (blue) contributions of the amino acid residues of the CX3CR1 isoforms (A) and cndG (B) in the four complexes calculated via MM-GBSA along the MD trajectories.

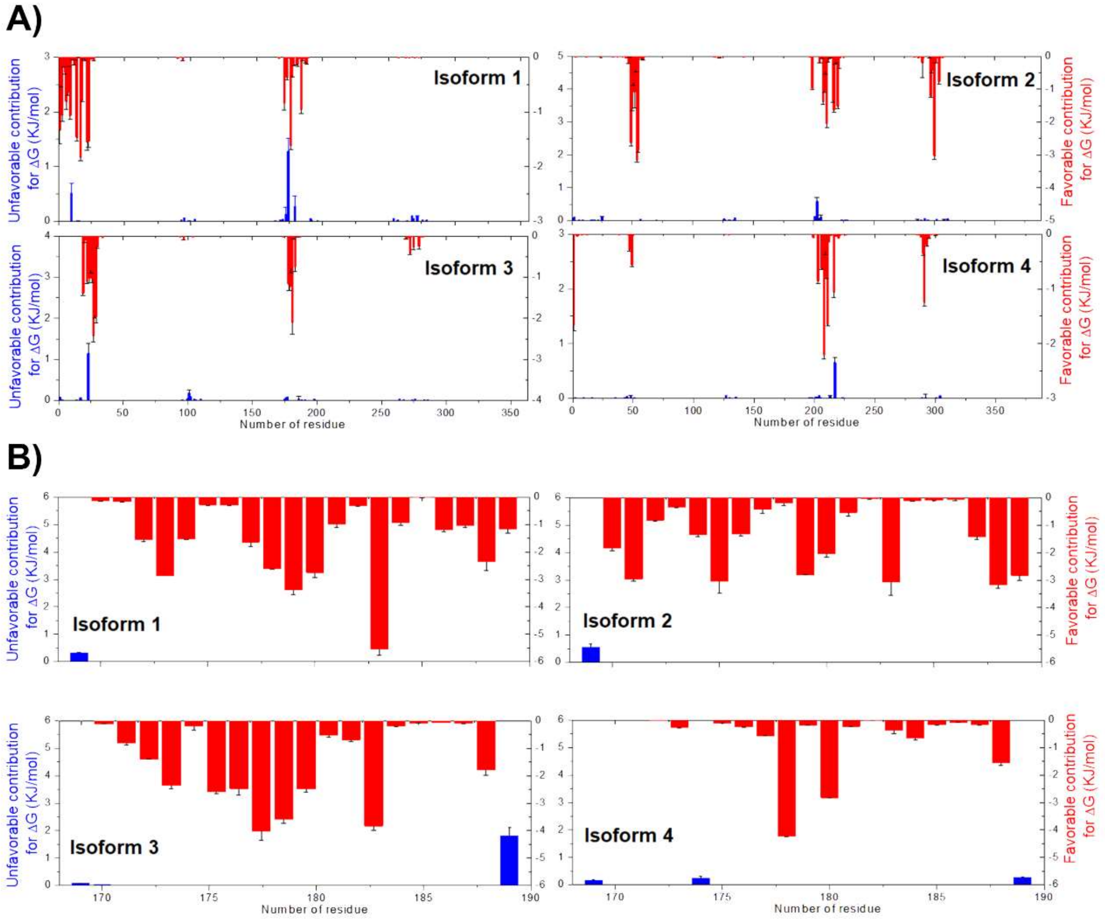

Figure S13. Sequence alignment of CX3CR1 and US28 receptors and comparison of its residues in the binding cavity. (A) Sequence alignment for the amino acid residue sequences of CX3CR1 (top, yellow) and US28 (bottom, red). (B and C) Residue numbering for CX3CR1 refers to its isoform 1. The key residues identified in the present study are highlighted in yellow in the residue sequence and denoted in the CX3CR1 isoform 1 structure in B while those reported in the literature for structural homologous US28 are highlighted in red in the sequence and indicated in this transmembrane receptor structure in C.

A

```
 11   NFEYDDLAEACYIGDIVVFGTVFLSIFYSVIFAIGLVGNLLVVFALTNSKKPKSVTDIYL  70
       F+YD+ A  C   D++           Y V+F  G +GN LV+F +T  ++ +   D+Y
 13   EFDYDEDATPCVFTDVLNQSKPVTLFLYGVVFLFGSIGNFLVIFTITWRRRIQCSGDVYF  72

 71   LNLALSDLLFVATLPFWTHYLINEKGLHNAMCKFTTAFFFIGFFGSIFFITVISIDRYLA  130
      +NLA +DLLFV TLP W  YL++    L +  C    TA F++  F S+ FIT I++DRY A
 73   INLAAADLLFVCTLPLWMQYLLDHNSLASVPCTLLTACFYVAMFASLCFITEIALDRYYA  132

131   IVLAANSMNNRTVQHGVTISLGVWAAAILVAAPQFMFTKQKENECLGDYPEVLQEIWPVL  190
      IV      M  R V+      S+  W  A+++A P FM    +K+N+C+ DY + L+  +P++
133   IVY----MRYRPVKQACLFSIFWWIFAVIIAIPHFMVVTKKDNQCMTDY-DYLEVSYPII  187
                                                        ECL2 Region

191   RNVETNFLGFLLPLLIMSYCYFRIIQTLFSCKNHKKAKAIKLILLVVIVFFLFWTPYNVM  250
       NVE      F++PL ++SYCY+RI + +    ++   K + +++++ VV+VF +FW PY++
188   LNVELMLGAFVIPLSVISYCYYRISRIVAVSQSRHKGRIVRVLIAVVLVFIIFWLPYHLT  247

251   IFLETLKLYDFF-PSCDMRKDLRLALSVTETVAFSHCCLNPLIYAFAGEKFRRYLYHLYG  309
      +F++TLKL   +    SC+   + L+ AL +TE++AF HCCLNPL+Y  F G KFR+ L+ L
248   LFVDTLKLLKWISSSCEFERSLKRALILTESLAFCHCCLNPLLYVFVGTKFRQELHCLLA  307

310   KCLAVLCGRSV   320
      +    L  R V
308   EFRQRLFSRDV   318
```

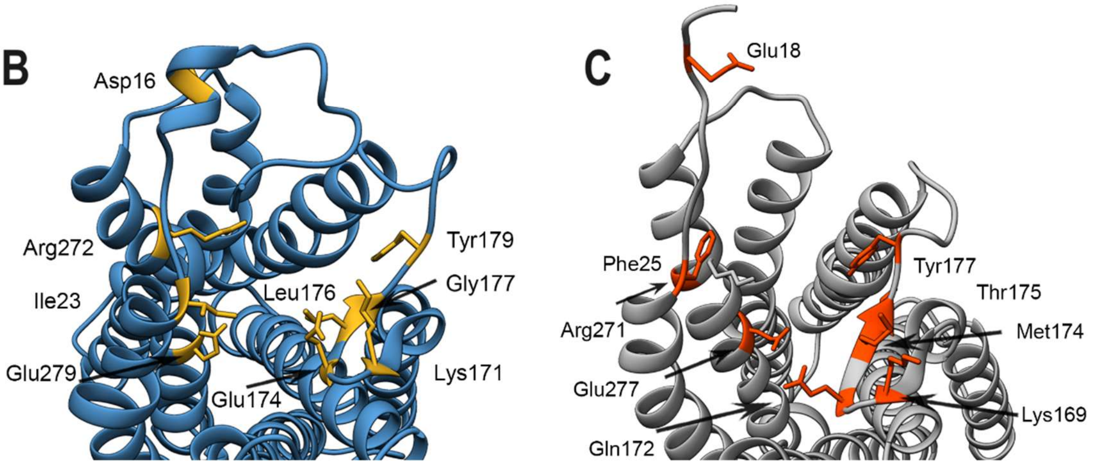

Figure S14. Multiple sequence alignment for homologous CX3CR1s of different species. The analyzed sequences were of *Homo sapiens* (isoform 1 of the present study), *Bos taurus*, *Chimpanzee*, *Mus musculus*, and *Sigmodon hispidus* from NCBI id ABS29268.1, ADD82806.1, NP_001139725.1, NP_034117.3, and QWL12666.1, respectively. The key residues identical and different to those in the *Homo sapiens* CX3CR1 sequence are highlighted in yellow and red, respectively. Sequence homology between CX3CR1 is denoted by asterisk (*), colon (:), and period (.). The asterisk indicates fully conserved residue, colon represents conservation between groups of strongly similar properties (scoring > 0.5), and period indicates conservation between groups of weakly similar properties (scoring ≤ 0.5).

```
Bos Taurus            MHTTLPESTSENFEYYDLAEVCDMGDIVALGTVFVVILYSLVFAFGLVGNLLVVFALINS 60
Homo Sapiens          -MDQFPESVTENFEYDDLAEACYIGDIVVFGTVFLSIFYSVIFAIGLVGNLLVVFALTNS 59
Chimpanzee            -MYQFPESVTENFEYDDLAEACSFGDIVVFGTVFLSIFYSVVFAIGLVGNLLVVFALTNS 59
Mus Musculus          MSTSFPELDLENFEYDDSAEACYLGDIVAFGTIFLSVFYALVFTFGLVGNLLVVLALTNS 60
Sigmodon Hispidus     MPTFFPGLDEENFEYDDSAEACNIGDIVAFGSIFMSILYSLVFAFGLVGNLLVVLALTNS 60
                          :*    ***** * **.* :****.:*::*: ::*:::*::*********:** **

Bos Taurus            QRSKSITDIYLLNLALSNLLFVATLPFWTHYVINEQGLHHATCKLITAFFFIGFFGGIFF 120
Homo Sapiens          KKPKSVTDIYLLNLALSDLLFVATLPFWTHYLINEKGLHNAMCKFTTAFFFIGFFGSIFF 119
Chimpanzee            KKPKSVTDIYLLNLALSDLLFVATLPFWTHYLINEKGLHNAMCKFTTAFFFIGFFGSIFF 119
Mus Musculus          RKPKSITDIYLLNLALSDLLFVATLPFWTHYLISHEGLHNAMCKLTTAFFFIGFFGGIFF 120
Sigmodon Hispidus     RKPKSITDIYLLNLALSDLLFVATLPFWVHYIISEEGLHNAVCKLTTAFFFIGFFGGIFF 120
                      :: **:***********:**********.**:*..:***:* **: **********.***

                                                                 ECL2 Region
Bos Taurus            ITVISVDRFLAIVLAANSMSNRTVQHGVTTSLGVWAAAILAATPQFMFTREKENECFGDY 180
Homo Sapiens          ITVISIDRYLAIVLAANSMNNRTVQHGVTISLGVWAAAILVAAPQFMFTKQKENECLGDY 179
Chimpanzee            ITVISIDRYLAIVLAANSMNNRTVQHGVTISLGVWAAAILVAAPQFMFTKQKENECLGDY 179
Mus Musculus          ITVISIDRYLAIVLAANSMNNRTVQHGVTISLGVWAAAILVASPQFMFTKRKDNECLGDY 180
Sigmodon Hispidus     ITVISIDRYLAIVLAANSMNNRTVQHGVTISLGVWAAAILVAAPQFMFTKRKENECLGDY 180
                      *****:**:**********.********* **********.*:******:.*:***:***

Bos Taurus            PEILQEIWPVILNTEINFLGFLLPLLIMSYCYFRIMQTLFSCKNHKKAKAIRLIFLVVVV 240
Homo Sapiens          PEVLQEIWPVLRNVETNFLGFLLPLLIMSYCYFRIIQTLFSCKNHKKAKAIKLILLVVIV 239
Chimpanzee            PEVLQEIWPVLRNVETNFLGFLLPLLIMSYCYFRIIQTLFSCKNHKKAKAIKLILLVVIV 239
Mus Musculus          PEVLQEMWPVLRNSEVNILGFALPLLIMSFCYFRIIQTLFSCKNRKKARAVRLILLVVFA 240
Sigmodon Hispidus     PEVLQEIWPVLRNSEVNILGFVLPLLIMSFCYFRIIQTLFSCKNHKKARAMRLILLVVVV 240
                      **:***:***: * * *:*** *******:*****:********:***:*::**:***..

Bos Taurus            FFLFWTPYNVMIFLQTLNLYDFFPKCDVKRDLKLAISVTETIAFSHCCLNPLIYAFAGEK 300
Homo Sapiens          FFLFWTPYNVMIFLETLKLYDFFPSCDMRKDLRLALSVTETVAFSHCCLNPLIYAFAGEK 299
Chimpanzee            FFLFWTPYNVMIFLETLKLYDFFPSCDMRKGLRLALSVTETVAFSHCCLNPLIYAFAGEK 299
Mus Musculus          FFLFWTPYNIMIFLETLKFYNFFPSCDMKRDLRLALSVTETVAFSHCCLNPFIYAFAGEK 300
Sigmodon Hispidus     FFLFWTPYNIVIFLETLKFYNFFPSCDTKRDLRLALSVTETVAFSHCCLNPLIYAFAGEK 300
                      *********::***:**::*:***.** ::.*:**:*****:*********:********

Bos Taurus            FRRYLYRLYRKCLAVLCCHPDHLSFSSSLSESQRSRRESVLSSNFTHYTSDGDASILL   358
Homo Sapiens          FRRYLYHLYGKCLAVLCGRSVHVDFSS--SESQRSRHGSVLSSNFTYHTSDGDALLLL   355
Chimpanzee            FRRYLYHLYGKCLAVLCGRSVHVDFSS--SESQRSRHGSVLSSNFTYHTSDGDASLLL   355
Mus Musculus          FRRYLGHLYRKCLAVLCGHPVHTGFSP---ESQRSRQDSILSSFTHY-TSEGDGSLLL   354
Sigmodon Hispidus     FRRYLRHLYRKCLSVLCGHPVHVSFTT---ESQRSRQDSILSSFTHY-TSDGEGSILL   354
                      ***** :** ***:*** :  * .*:    ******: *:***   : **:*:. :**
```

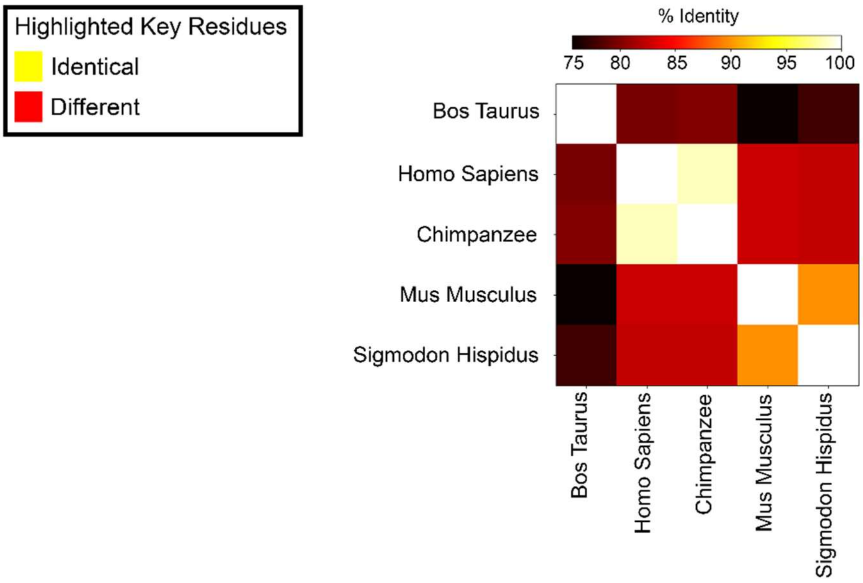

Table S1. Values of the percentages of the ψ and ϕ torsional angles of the residues of the structural models of the CX3CR1 isoform 1 determined from Ramachandran plot.

Residues	RoseTTAFold	trRosetta	AlphaFold	Phyre2	I-tasser
in most favored regions	92.7%	90.9%	88.1%	85.3%	77.1%
in additional allowed regions	6.1%	7.6%	9.5%	10.1%	17.7%
in generously allowed regions	0.6%	0.3%	0.6%	2.4%	3.4%
in disallowed regions	0.6%	1.2%	1.8%	2.2%	1.8%

Table S2. Percentage of persistence of hydrogen bonds > 10% for the structural models of the complexes of cndG with CX3CR1 isoforms 1, 2, 3, and 4 from ClusPro server. To differentiate from the protein, cndG residues are marked with an asterisk. The number in parentheses corresponds to the reference numbering of isoform 1.

Donor	Atom	Acceptor	Atom	%Persistence
		**Isoform 1**		
Cys173*	N	Glu6	OE1	70.484
Cys173*	N	Glu6	OE2	66.418
Ser174*	N	Glu6	OE1	59.872
Ser174*	N	Glu6	OE2	33.516
Ile175*	N	Glu6	OE1	30.689
Ile175*	N	Glu6	OE2	58.419
Ser177*	OG	Glu254	OE1	49.047
Ser177*	OG	Glu254	OE2	48.660
Asn178*	ND2	Glu254	OE1	43.114
Asn178*	ND2	Glu254	OE2	43.674
Asn178*	ND2	Glu279	OE1	15.051
Asn178*	ND2	Glu279	OE2	16.291
Asn179*	N	Ser276	OG	49.847
Asn179*	ND2	Glu279	OE1	39.181
Asn179*	ND2	Glu279	OE2	43.674
Arg188*	N	Gln3	OE1	23.610
Arg188*	NH1	Cys21	O	28.316
Arg188*	NH2	Gln3	OE1	14.558
Arg188*	NH2	Cys21	O	25.757
Ile189*	O	Glu6	OE1	63.991
Ile189*	O	Glu6	OE2	34.902
Gln3	NE2	Ser174*	OG	22.517
Gln3	NE2	Cys186*	O	19.104
Gln184	NE2	Phe170*	O	38.475
Arg191	NH2	Cys176*	O	10.439
Arg191	NH2	Ser177*	OG	15.065
Arg191	NH2	Asn178*	OD1	12.985
Arg272	NH1	Ser177*	OG	31.809
		**Isoform 2**		
Asn169*	ND2	Leu49 (17)	O	16.238
Asn169*	ND2	Glu51 (19)	O	27.836
Asn169*	ND2	Cys53 (21)	O	44.314
Phe170*	N	Leu49 (17)	O	37.182

Asn178*	ND2	Tyr122 (90)	O	24.623
Asn178*	ND2	Cys207 (175)	O	10.092
Thr181*	OG1	Tyr54 (22)	OH	40.288
Arg188*	NH1	Tyr211 (179)	OH	10.932
Tyr122 (90)	OH	Ser174*	O	20.677
**Isoform 3**				
Ser177*	OG	Leu98 (91)	O	81.802
Asn178*	N	Tyr97 (90)	O	66.378
Asn179*	ND2	Arg279 (272)	O	22.357
Asn179*	ND2	Ser283 (276)	OG	20.837
Arg188*	NE	Glu181 (174)	OE1	11.918
Arg188*	NE	Glu181 (174)	OE2	10.012
Arg188*	NH1	Glu101 (94)	O	11.998
Arg188*	NH2	Glu101 (94)	O	11.079
Ile189*	O	Glu101 (94)	OE1	12.945
Ile189*	O	Glu101 (94)	OE2	17.598
Tyr29 (22)	OH	Pro172*	O	13.811
Arg279 (272)	NE	Asn178*	OD1	24.170
Arg279 (272)	NH2	Asn178*	OD1	11.159
Ser283 (276)	OG	Asn178*	OD1	11.452
**Isoform 4**				
Asn169*	N	Thr287 (255)	OG1	13.478
Asn169*	N	Asp292 (260)	OD1	11.385
Asn169*	ND2	Tyr211 (179)	OH	19.051
Asn169*	ND2	Asp292 (260)	OD1	18.491
Asn169*	ND2	Asp292 (260)	OD2	21.810
Ser177*	OG	Glu311 (279)	OE1	39.821
Ser177*	OG	Glu311 (279)	OE2	14.825
Asn178*	N	Glu311 (279)	OE1	26.410
Asn178*	N	Glu311 (279)	OE2	23.197
Asn178*	ND2	Tyr70 (38)	OH	11.159
Asn178*	ND2	Thr310 (278)	O	24.023
Asn178*	ND2	Glu311 (279)	OE1	15.665
Asn178*	ND2	Glu311 (279)	OE2	15.278
Asn179*	ND2	Thr227 (195)	OG1	46.434
Asn179*	ND2	Tyr279 (247)	OH	75.497
Thr181*	OG1	Asn224 (192)	OD1	10.999
Thr181*	OG1	Glu226 (194)	OE2	45.661
Thr181*	OG1	Thr227 (195)	OG1	20.184
Lys187*	NZ	Gly209 (177)	O	45.421
Lys187*	NZ	Asp210 (178)	OD1	13.545
Lys187*	NZ	Asp210 (178)	OD2	14.371
Lys187*	NZ	Tyr211 (179)	OH	17.651
Arg188*	N	Cys207 (175)	O	96.121
Ile189*	N	Glu206 (174)	OE1	32.742
Ile189*	N	Glu206 (174)	OE2	65.298
Gly209 (177)	N	Cys186*	O	88.748
Tyt211 (179)	OH	Asn169*	OD1	21.064
Asn228 (196)	ND2	Asn179*	OD1	13.012
Arg300 (268)	NH1	Asn169*	OD1	21.370

Arg300 (268)	NH2	Asn169*	OD1	15.118
Arg304 (272)	NE	Ser174*	OG	27.076

Table S3. Percentage of persistence of hydrogen bonds > 10% for the structural models of the complexes of cndG with CX3CR1 isoforms 1, 2, 3, and 4 from HADDOCK server. To differentiate from the protein, cndG residues are marked with an asterisk. The number in parentheses corresponds to the reference numbering of isoform 1.

Donor	Atom	Acceptor	Atom	%Persistence
		**Isoform 1**		
Cys173*	N	Glu10	OE1	41.861
Cys173*	N	Glu10	OE2	41.381
Ser174*	N	Glu10	OE1	45.181
Ser174*	N	Glu10	OE2	43.141
Ser174*	OG	Glu10	OE1	33.062
Ser174*	OG	Glu10	OE2	33.622
Ser177*	OG	Glu6	OE1	12.718
Ser177*	OG	Glu6	OE2	11.678
Asn178*	N	Glu6	O	21.837
Asn178*	ND2	Glu6	OE1	14.531
Asn178*	ND2	Glu6	OE2	14.865
Asn178*	ND2	Gly177	O	23.850
Asn178*	ND2	Tyr179	OH	44.394
Asn179*	ND2	Lys171	O	11.812
Asn179*	ND2	Glu172	OE1	17.051
Asn179*	ND2	Glu172	OE2	16.318
Asn179*	ND2	Glu174	OE1	23.863
Asn179*	ND2	Glu174	OE2	31.276
Thr181*	N	Glu174	OE1	44.967
Thr181*	N	Glu174	OE2	18.371
Thr181*	OG1	Glu172	OE1	15.985
Thr181*	OG1	Glu172	OE2	16.984
Thr181*	OG1	Glu174	OE1	49.740
Thr181*	OG1	Glu174	OE2	45.301
Trp183*	NE1	Asp2	O	18.011
Met1	N	Trp183*	O	11.972
Met1	N	Cys186*	O	18.691
Gln3	NE2	Pro180*	O	20.517
Lys171	NZ	Ser177*	OG	10.985
Ly171	NZ	Ser177*	O	12.265
Lys171	NZ	Asn178*	OD1	20.677
Lys171	NZ	Asn178*	O	23.664
Tyr179	N	Asn178*	OD1	12.185
Gln184	NE2	Ser174*	O	11.812
		**Isoform 2**		
Ser177*	N	Tyr211 (179)	OH	73.350
Ser177*	OG	Tyr211 (179)	OH	26.010
Asn179*	ND2	Gly209 (177)	O	76.496
Asn179*	ND2	Asp210 (178)	OD1	26.690

Asn179*	ND2	Asp210 (178)	OD2	25.117
Ile189*	N	Leu49 (17)	O	44.514
Lys203 (171)	NZ	Thr181*	OG1	12.425
Gln216 (184)	NE2	Asn169*	O	19.784
Arg300 (268)	NH2	Cys176*	O	21.904
Arg304 (272)	NH1	Asn178*	OD1	22.064
Arg304 (272)	NH2	Asn178*	OD1	18.264
**Isoform 3**				
Ser174*	N	Asp23 (16)	OD1	18.144
Ser174*	N	Asp23 (16)	OD2	15.651
Ser174*	OG	Asp23 (16)	OD1	16.918
Ser174*	OG	Asp23 (16)	OD2	15.905
Ser177*	N	Glu181 (174)	OE1	54.086
Ser177*	N	Glu181 (174)	OE2	26.530
Ser177*	OG	Asp22 (15)	OD1	14.371
Ser177*	OG	Asp22 (15)	OD2	12.452
Asn178*	N	Glu181 (174)	OE1	59.472
Asn178*	N	Glu181 (174)	OE2	38.235
Asn178*	ND2	Glu181 (174)	OE1	19.584
Asn178*	ND2	Glu181 (174)	OE2	23.104
Asn179*	N	Glu181 (174)	OE1	55.073
Asn179*	N	Glu181 (174)	OE2	25.983
Asn179*	ND2	Glu101 (94)	OE1	40.968
Asn179*	ND2	Glu101 (94)	OE2	43.381
Thr181*	OG1	Glu101 (94)	OE1	23.264
Thr181*	OG1	Glu101 (94)	OE2	26.810
Arg188*	NH1	Asp23 (16)	O	18.011
Ile189*	O	Asp23 (16)	OD1	20.117
Ile189*	O	Asp23 (16)	OD2	22.424
Cys28 (21)	N	Asn178*	OD1	11.398
Cys28 (21)	N	Asn178*	O	35.155
Tyr29 (22)	N	Asn178*	O	14.611
Lys178 (171)	NZ	Ser174*	O	11.425
Lys178 (171)	NZ	Ser177*	OG	17.638
Asn180 (173)	ND2	Ile175*	O	10.972
Asn180 (173)	ND2	Asn179*	OD1	25.130
**Isoform 4**				
Ser174*	OG	Glu217 (185)	OE1	33.982
Ser174*	OG	Glu217 (185)	OE2	31.916
Asn178*	N	Gln216 (184)	OE1	21.117
Asn178*	ND2	Gly209 (177)	O	96.947
Lys203 (171)	NZ	Asn178*	OD1	34.782
Tyr211 (179)	N	Asn178*	OD1	68.911

Table S4. The most significant contributions of binding free energy via MM-GBSA of the residues of isoform 1, 2, 3, and 4 for the structural models of the CX3CR1/cndG complexes from the ClusPro server. The values in brackets denote the value of average plus standard deviation calculated on the contribution of all amino acid residues. The most significant energy contributions correspond to values greater than that in the brackets. The number in parentheses corresponds to the reference numbering of isoform 1.

Residue	Favorable energy	Residue	Unfavorable energy
	**Isoform 1**		
	**[<-0.5732) kJ/mol]**		**[>0.3003) kJ/mol]**
Asp2	-0.75	Glu6	4.13
Gln3	-3.49	Glu254	1.28
Phe4	-1.65		
Ser7	-0.81		
Cys21	-0.99		
Ile23	-2.16		
Ile26	-1.58		
Val27	-1.75		
Trp87	-1.51		
Tyr90	-1.92		
Leu91	-1.92		
Glu174	-0.86		
Leu176	-2.15		
Tyr179	-1.66		
Pro180	-0.57		
Glu181	-1.72		
Leu183	-0.74		
Gln184	-1.73		
Trp187	-2.34		
Pro188	-1.48		
Arg191	-0.69		
Arg272	-2.23		
Leu273	-0.65		
Ser276	-1.27		
	**Isoform 2**		
	**[<-0.5204 kJ/mol]**		**[>0.02973 kJ/mol]**
Trp24 (–8)	-1.67	Met1 (–31)	0.1
Leu49 (17)	-2.02	Arg2 (–30)	0.14
Ala50 (18)	-0.9	Tyr54 (22)	0.12
Glu51 (19)	-1.13	Lys201 (169)	0.06
Ala52 (20)	-1.64	Lys203 (171)	0.08
Ile55 (23)	-4.1	Glu204 (172)	0.03
Ile58 (26)	-0.52	Glu206 (174)	0.29
Val59 (27)	-1.22	Lys289 (257)	0.07
Trp119 (87)	-1.14	Ser296 (264)	0.03
Tyr122 (90)	-1.52	Lys301 (269)	0.04
Leu123 (91)	-0.91	Ser308 (276)	0.03
Phe199 (167)	-2.29	Glu311 (279)	0.09
Cys207 (175)	-0.53		
Leu208 (176)	-4.62		

Gly209 (177)	-0.53		
Tyr211 (179)	-1.38		
Trp219 (187)	-0.65		
Pro220 (188)	-1.16		
**Isoform 3**			
	**[<-0.4363 kJ/mol]**		**[>0.0163 kJ/mol]**
Ala27 (20)	-0.94	Met1 (–6)	0.08
Cys28 (21)	-1.18	Ser3 (–4)	0.01
Tyr29 (22)	-3.41	Asn18 (11)	0.01
Ile30 (23)	-3.26	Asp32 (25)	0.01
Val34 (27)	-1.58	His105 (98)	0.02
Tyr97 (90)	-1.1	Lys110 (103)	0.04
Leu98 (91)	-0.62	Lys176 (169)	0.04
Lys178 (171)	-0.48	Gln177 (170)	0.06
Asn180 (173)	-0.48	Gln191 (184)	0.01
Glu181 (174)	-2.43	Arg198 (191)	0.05
Cys182 (175)	-0.63	Asn199 (192)	0.01
Leu183 (176)	-2.21	Glu261 (254)	0.02
Cys272 (265)	-0.47	Lys264 (257)	0.05
Lys276 (269)	-0.57	Ser283 (276)	0.19
Arg279 (272)	-1.91	Glu286 (279)	0.05
Leu280 (273)	-0.7		
**Isoform 4**			
	**[<-0.6451 kJ/mol]**		**[>0.0574 kJ/mol]**
Arg2 (–30)	-2.48	Met1 (–31)	0.45
Leu49 (17)	-2.46	Glu6 (–26)	0.25
Ala50 (18)	-0.72	Asp47 (15)	0.08
Cys53 (21)	-2.25	Lys203 (171)	0.06
Tyr54 (22)	-0.86	Glu226 (194)	0.41
Trp119 (87)	-1.71	Thr227 (195)	0.42
Phe141 (109)	-1.97	Lys289 (257)	0.07
Phe142 (110)	-1.68		
Gln196 (164)	-1.47		
Phe199 (167)	-2.09		
Thr200 (168)	-1.34		
Glu206 (174)	-1.46		
Cys207 (175)	-2.46		
Leu208 (176)	-3.01		
Gly209 (177)	-3.13		
Asp210 (178)	-1.52		
Tyr211 (179)	-1.09		
Arg223 (191)	-1.86		
Tyr279 (247)	-0.86		
Ile283 (251)	-1.9		
Glu286 (254)	-0.68		
Thr287 (255)	-0.72		
Leu290 (258)	-1.24		
Tyr291 (259)	-1.85		
Asp292 (260)	-0.67		
Arg304 (272)	-1.34		

Leu307 (275)	-1.83

Table S5. The most significant contributions of binding free energy via MM-GBSA of the residues of isoform 1, 2, 3, and 4 for the structural models of the CX3CR1/cndG complexes from the HADDOCK server. The values in brackets denote the value of average plus standard deviation calculated on the contribution of all amino acid residues. The most significant energy contributions correspond to values greater than that in the brackets. The number in parentheses corresponds to reference numbering of isoform 1.

Residue	Favorable energy	Residue	Unfavorable energy
**Isoform 1**			
	**[<-0.3012 kJ/mol]**		**[>0.09002 kJ/mol]**
Met1	-1.33	Glu10	0.51
Asp2	-0.51	Glu172	0.12
Gln3	-1.05	Glu174	1.28
Glu6	-0.81	Tyr279	0.27
Ser7	-0.65	Arg268	0.1
Thr9	-1.07	Arg272	0.09
Tyr14	-1.46		
Leu17	-1.82		
Ala18	-0.78		
Tyr22	-1.55		
Ile23	-1.53		
Lys171	-0.84		
Asn173	-0.37		
Leu176	-1.6		
Gly177	-0.34		
Gln184	-0.85		
**Isoform 2**			
	**[<-0.4781 kJ/mol]**		**[>0.0409 kJ/mol]**
Leu49 (17)	-2.63	Met1 (–31)	0.08
Aal50 (18)	-1.59	Arg2 (–30)	0.04
Glu51 (19)	-0.81	Arg25 (–7)	0.11
Ala52 (20)	-1.08	Asp57 (25)	0.04
Tyr54 (22)	-3.14	Glu126 (94)	0.05
Ile55 (23)	-2.83	Lys135 (103)	0.07
Phe199 (167)	-0.97	Lys201 (169)	0.11
Leu208 (176)	-1.36	Lys203 (171)	0.59
Gly209 (177)	-1.04	Glu204 (172)	0.04
Asp210 (178)	-0.47	Glu206 (174)	0.1
Tyr211 (179)	-2.04	Glu286 (254)	0.04
Gln216 (184)	-1.34	Asp292 (260)	0.05
Glu217 (185)	-1.63	Asp302 (270)	0.04
Pro220 (188)	-1.52	Glu311 (279)	0.05
Cys297 (265)	-1.2		
Arg300 (268)	-3.02		
Arg304 (272)	-0.76		
**Isoform 3**			
	**[<-0.3412 kJ/mol]**		**[>0.0705 kJ/mol]**

Phe19 (12)	-1.38	Met1 (–6)	0.07
Asp22 (15)	-1.09	Asp23 (16)	1.14
Leu24 (17)	-1.02	Glu101 (94)	0.18
Ala25 (18)	-0.84	Lys102 (95)	0.09
Glu26 (19)	-1.03	Gln177 (170)	0.07
Ala27 (20)	-2.41		
Cys28 (21)	-1.92		
Tyr29 (22)	-1.96		
Lys178 (171)	-1.16		
Glu179 (172)	-1.23		
Asn180 (173)	-0.78		
Glu181 (174)	-2.09		
Leu183 (176)	-0.74		
Cys272 (265)	-0.41		
**Isoform 4**			
	**[<-0.2383 kJ/mol]**		**[>0.0386 kJ/mol]**
Met1 (–31)	-1.66	Glu45 (13)	0.03
Asp47 (15)	-0.28	Asp48 (16)	0.04
Leu49 (17)	-0.56	Lys127 (95)	0.05
Lys203 (171)	-0.86	Glu204 (172)	0.04
Glu206 (174)	-0.63	Glu213 (181)	0.04
Leu208 (176)	-2.2	Glu217 (185)	0.65
Gly209 (177)	-0.32	Arg304 (272)	0.04
Asp210 (178)	-0.79		
Tyr211 (179)	-1.64		
Gln216 (184)	-1.05		
Leu290 (258)	-0.36		
Tyr291 (259)	-1.24		

Table S6. The most significant contributions of binding free energy via MM-GBSA of the residues of cndG for the four structural models of the CX3CR1/cndG complexes from the ClusPro server. The values in brackets denote the value of average plus standard deviation calculated on the contribution of all amino acid residues. The most significant energy contributions correspond to values greater than that in the brackets.

Residue cndG/ isoform1	energy [-4.6912] kJ/mol	Residue cndG/ isoform2	energy [-3.2976] kJ/mol	Residue cndG/ isoform3	energy [-3.1460] kJ/mol	Residue cndG/ isoform4	energy [-4.2629] kJ/mol
Cys173	-4.71	Pro180	-3.65	Pro180	-3.23	Pro180	-5.16
Ile175	-6.63	Trp183	-5.47	Trp183	-5.98	Thr181	-4.38
Trp183	-7.73					Ile185	-5.23
						Ile189	-4.75

Table S7. The most significant contributions of binding free energy via MM-GBSA of the residues of cndG for the four structural models of the CX3CR1/cndG complexes from the HADDOCK server. The values in brackets denote the value of average plus standard deviation calculated on the contribution of all amino acid residues. The most significant energy contributions correspond to values greater than that in the brackets.

Residue cndG/ isoform1	energy [-2.9214] kJ/mol	Residue cndG/ isoform2	energy [-2.6231] kJ/mol	Residue cndG/ isoform3	energy [-2.9178] kJ/mol	Residue cndG/ isoform4	energy [-1.7827] kJ/mol
Asn179	-3.38	Val171	-2.96	Asn178	-4.01	Asn178	-4.23
Trp183	-5.54	Ile175	-3.04	Asn179	-3.59	Pro180	-2.83
		Asn179	-2.81	Trp183	-3.83		
		Trp183	-3.07				
		Arg188	-3.18				
		Ile189	-2.83				
